# Sequence analysis on the information of folding initiation segments in ferredoxin-like fold proteins

**DOI:** 10.1186/1472-6807-14-15

**Published:** 2014-05-23

**Authors:** Masanari Matsuoka, Takeshi Kikuchi

**Affiliations:** 1 Department of Bioinformatics, College of Life Sciences, Ritsumeikan University, 1-1-1 Nojihigashi, Kusatsu, Shiga 525-8577, Japan; 2 Japan Society for the Promotion of Science (JSPS), Tokyo, Japan

**Keywords:** Folding initiation segment prediction, Sequence analysis, Inter-residue average distance statistics, Evolutionarily conserved folding, Ribosomal protein S6, Procarboxypeptidase A2, U1A Spliceosomal protein, mt-Acylphosphatase

## Abstract

**Background:**

While some studies have shown that the 3D protein structures are more conservative than their amino acid sequences, other experimental studies have shown that even if two proteins share the same topology, they may have different folding pathways. There are many studies investigating this issue with molecular dynamics or Go-like model simulations, however, one should be able to obtain the same information by analyzing the proteins’ amino acid sequences, if the sequences contain all the information about the 3D structures. In this study, we use information about protein sequences to predict the location of their folding segments. We focus on proteins with a ferredoxin-like fold, which has a characteristic topology. Some of these proteins have different folding segments.

**Results:**

Despite the simplicity of our methods, we are able to correctly determine the experimentally identified folding segments by predicting the location of the compact regions considered to play an important role in structural formation. We also apply our sequence analyses to some homologues of each protein and confirm that there are highly conserved folding segments despite the homologues’ sequence diversity. These homologues have similar folding segments even though the homology of two proteins’ sequences is not so high.

**Conclusion:**

Our analyses have proven useful for investigating the common or different folding features of the proteins studied.

## Background

Clarifying how a protein folds into its unique 3D structure is a very significant yet unsolved problem in molecular biophysics and bioinformatics [1]. In particular, some recent experimental studies have revealed that proteins sharing the same topology can take different folding pathways [2-10].

Ferredoxin-like fold proteins are well-known proteins that fold via different folding pathways. Their topology is composed of 2 α helices and 4 β strands, and the secondary structure arrangement seems similar to the β/α/β triad motif in flavodoxin [11,12] or TIM-barrel proteins [13]. However, the connectivity of the secondary structures differs. While flavodoxin or TIM-barrel proteins have a parallel β sheet, ferredoxin-like proteins have an anti-parallel β sheet. Therefore, it is hard to explain the ferredoxin-like proteins’ folding behavior only with the formation of β/α/β triads and the interaction among subdomains as in the case of flavodoxin, even if it is true that most of the hydrophobic contacts are composed of Ile, Leu and Val residues as reported in the literature [14].

**Figure 1:**
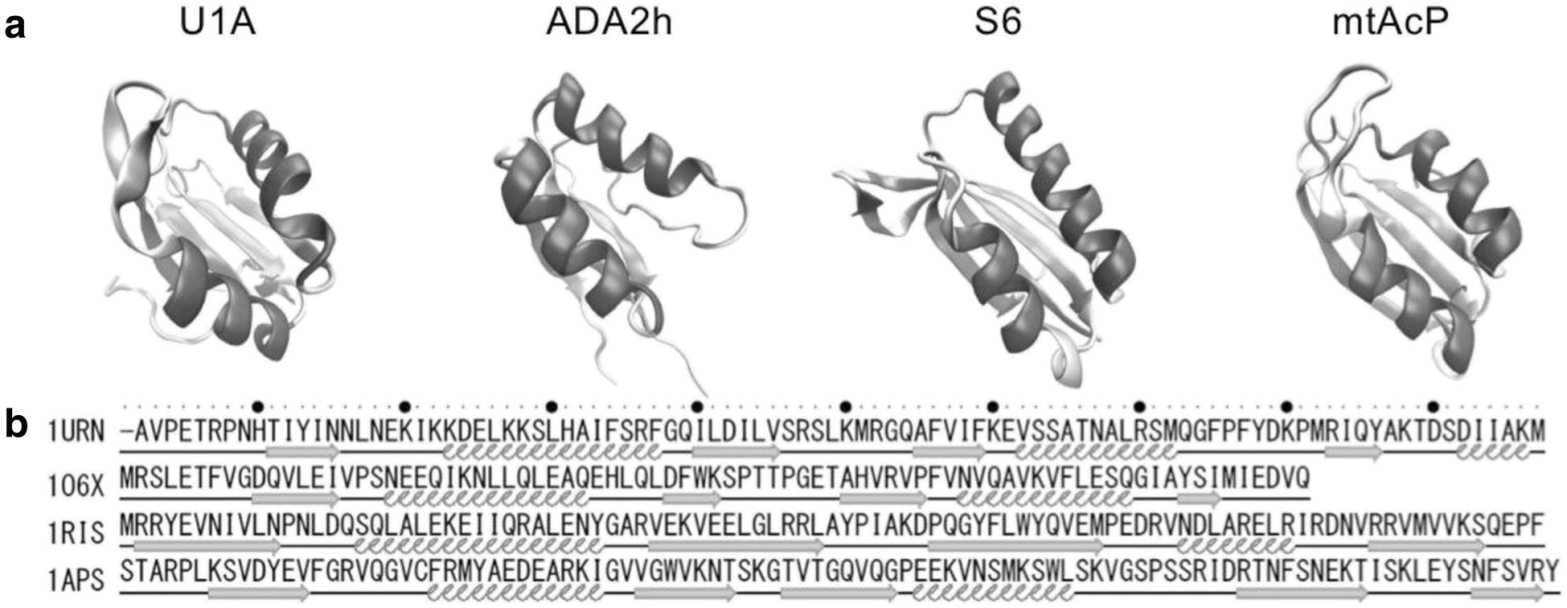
**Proteins treated in this study. (a)** Tertiary structures of the study proteins obtained from the Protein Data Bank [18]. Their names are shown above the structures. α helices and β strands are represented with helices and arrows, respectively. These protein models are created by Visual Molecular Dynamics 1.9.1 [19]. **(b)** Amino acid sequences of the study proteins. The filled circles above them denote the location of decadal number residues. Arrows and helices below the sequences indicate the location of secondary structures.

Ferredoxin-like fold proteins are relatively small as shown in Figure 1, but they have interesting features in the structural transformation from denatured states to transit or native states. For example, one ferredoxin-like protein called ribosomal protein S6 contains two overlapping foldons, which fold cooperatively, located at different termini, and the overlapping makes this protein fold in a two-state manner as reported by Haglund et al. [15]. However, other proteins such as U1A spliceosomal protein or procarboxypeptidase A2 fold via the N- or C-terminal foldon as reported by Ternström et al. [16] or Villegas et al. [17], respectively.

If all the information related to the formation of 3D structures is encoded in the amino acid sequences, we should be able to decode these sequence differences to obtain their folding features. Still, this is a challenging task. How the folding mechanism of a protein is coded in the amino acid sequence information remains an important issue to be clarified.

There are some bioinformatics approaches for predicting some aspects of folding mechanisms, like folding rate, from the amino acid sequence [20-27]. Nevertheless, it is still difficult to extract more detailed information on the folding mechanism, that is, how each protein folds.

The fact that the topologically equivalent proteins do not always fold via the same folding pathway leads us to the question of whether evolutionarily related proteins really have common folding properties. Evolutionarily related proteins have been observed to be possible to fold via different folding pathways. For example, in spite of the fact that PDZ2 and PDZ3, both of which contain mainly β sheets, are evolutionarily related and have more than 30% sequence identity, they do not share the same folding mechanism, at least in the early stage of folding [28]. On the other hand, fibronectin and titin, which are evolutionarily unrelated proteins but have the same topology, share the folding mechanism involving four key residues and their peripheral residues [29]. There are also some other studies focusing on the differences in the folding of topologically equivalent proteins [3]; yet, these experiments were performed only for several proteins of each topology and were not applied to a whole family.

In this study, we aim to decode such information for well-studied ferredoxin-like fold [30] proteins by analyzing their amino acid sequences, not only with the preiously mentioned bioinformatics approaches but also with our own analyses. The methods we apply here are homologous sequence search, phylogenetic analysis and sequence-based analyses by means of inter-residue average distance statistics.

The methods, which are based on the inter-residue average distance statistics [31,32] using the amino acid sequences as input, have so far provided valuable information on the initial folding segments that play crucial roles in the structural formation in the cases of lysozyme, leghemoglobin, fatty acid-binding protein, azurin, and two ancient TIM-barrel proteins [33-36]. We also apply our methods to some evolutionarily related proteins of four ferredoxin-like fold proteins to examine whether evolutionarily related proteins have common folding properties.

## Methods

### Proteins treated in this study

The proteins treated in this study are U1A spliceosomal protein (U1A) [PDB: 1URN] [37], procarboxypeptidase A2 (ADA2h) [PDB: 1O6X] [38], ribosomal protein S6 (S6) [PDB: 1RIS] [39], and muscle-type acylphosphatase (mtAcP) [PDB: 1APS] [40] as shown in Figure 1. These were selected through the Protein Folding Database 2.0, [41] which provides experimental folding data on proteins [15-17,42]. We call these proteins our “study proteins”. The amino acid sequences of these proteins were obtained from the structured region in their PDB files. Their sequence identities are quite low, ranging from 11 to 23%. Many experimental studies on these proteins have been performed with respect to the ferredoxin-like fold, and some of these studies suggest the existence of different folding segments [15-17,42].

### Inter-residue average distance statistics

To prepare the statistics for our methods, we calculate the average distance and its standard deviation for each inter-residue pair in 42 various proteins, considering the amino acid types and the sequence separation. For the sequence separation, we simplify the sequence separations k: 1 ~ 8, 9 ~ 20, 21 ~ 30, 31 ~ 40… in terms of the ranges M: 1, 2, 3, 4…, respectively. The 42 representative proteins were carefully chosen so as not to be biased towards some specific structures and have been confirmed to extract the regions corresponding to the structural domains. Because our analysis results are strongly affected by the particular protein datasets used, we chose not to alter the datasets based on the analysis results and to instead use the same datasets as in the first paper on ADM (Ref. [31]) to allow for comparability. We present the 42 proteins (Additional file 1: Table S3).

### Average Distance Map analysis

The regions predicted by Average Distance Map (ADM) analysis correspond to the regions that tend to be compact in their 3D structure. We believe that these compact regions might be structured in the early stage of folding.

The ADM analysis itself is a method for predicting the location of possible structural units in a protein by analyzing a predicted contact map [31] based on inter-residue average distance statistics. This map is referred to as an ADM and is used to extract standard structural units, such as structural domains or compact regions. In the ADM, any pair of residues with smaller average distance is considered to be in contact more than the other pairs, so the segment with many such pairs is considered to form a structural unit like folding segments by mechanisms such as hydrophobic collapse. These segments are automatically extracted by analyzing the ADM (as explained in the following text).

In the current study, we use this method to extract compact regions (not structural domains). For each compact region, the strength as a predicted folding segment is expressed as an η value. The η value tends to be higher if the corresponding compact regions have many contacts within the region or with the other regions including non-local areas (for more details, see Refs. [31,43] or [Additional file 1]). Contact density has been reported to correctly identify the nucleating subdomains in T4 lysozyme and interleukin-1β [12]. Studies on flavodoxin-like proteins also suggest a relationship betrween contact density and the folding rate of the corresponding area by showing that low contact density leads to structuring late in folding [44]. Thus, it would be reasonable to consider the regions with many contacts (a high η value) as the region structured in the early stage of folding.

Finally, all high-η-value units which do not overlap with other high-η-value units are designated as predicted folding segments, except for units that cover the whole sequence. When a predicted folding segment covers 70 to 100% of the whole sequence length, we conduct an additional search for folding segments overlapping in this unit. Because we could find only two folding segments for each protein, in this study, we call the segment with the higher η value the primary segment and the other, the auxiliary segment.

In Additional file 1: Figure S5, we compare the ADMs of the ferredoxin-like proteins with the actual contact map constructed based on the contacts defined later.

### Comparison of the regions predicted by two ADMs

**Figure 2:**
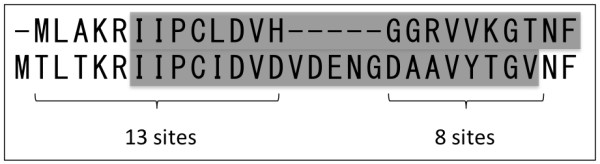
**Example of two sample aligned sequences from multiple alignment.** A region in gray corresponds to a compact region predicted by the ADM. Because there are 23 sites that have no gap in either sequence and there are 21 sites that are commonly included in or excluded from the predicted compact regions, 21/23 ~ 91.3% is the ADM similarity for this example.

Suppose that a multiple alignment of homologous sequences in a ferredoxin-like protein is obtained. Since it is convenient to define the similarity of location predicted by any two ADMs in the multiple alignment, we define the similarity as follows: First, two sequences are chosen from the aligned sequences, as shown in Figure 2. Second, all sites with a gap in either one or both sequences are removed. Here, “site” refers to the common sequential number in the multiple alignment. Finally, the number of sites that are commonly included in or excluded from the regions predicted by the ADMs for two given sequences is calculated. The ratio of this number to the number of all the non-gapped sites is defined as the similarity of location predicted by the two ADMs.

It should be noted that the similarity calculated by this procedure does not take η values and gapped sites into account: the present method is therefore not suitable in cases where the sequences in an alignment show large gaps. Having said that, the multiple alignments using the sequences obtained in this study contain few small gaps. For this reason, we can apply this definition of the similarity to the present results.

### F-value analysis

We performed additional analyses to determine the location where initial folding events, such as hydrophobic collapse, happen [32]. Using other statistical potentials like Miyazawa-Jernigan [45] and Skolnick [46] do may return similar information, but it makes difficult to interpret the results with ADM. Because there is not only the average distance but also the standard deviation of distance in inter-residue average distance statistics, we expect that the potential used in our F-value analysis to better reproduce the dynamics of the denatured state ensemble compared to the potential based on the contact energy. In F-value analysis, we use the Cα bead model to represent a protein’s structure, as well as the Metropolis Monte Carlo method with the potential energy *ϵ*_*i,j*_ derived from average distance $\bar{r_{i,j}}$ and its standard deviation *σ*_*i,j*_. The bond and dihedral angles of the initial conformation are randomly selected. The movement in the simulation is done as follows: The bond angle between the residue i and i + 1 is bent and rotated randomly from -10 to 10° followed by the Metropolis judgment to decide if the new conformation is acceptable or not. Within a step, i = 1…N-1 is performed, that is, all the bond angles are altered and judged.

The probability density with the potential energy between two residues, *P*(*ϵ*_*i,j*_), is hypothesized as being equivalent to the probability density based on the standard Gaussian distribution calculated with its average distance and standard deviation, $\rho \left(\bar{r_{i,j}},\sigma_{i,j}\right)$, as follows:

(1) $$P\left(\epsilon_{i,j}\right)=\rho \left(\bar{r_{i,j}},\sigma_{i,j}\right)$$

Where this equation can be expressed by equation (2);

(2) $$\frac{\exp\left(-\frac{\epsilon_{i,j}}{\mathrm{kT}}\right)}{Z}=\frac{1}{\sqrt{2\pi }\sigma_{i,j}}\exp\left\{-\frac{{\left(r_{i,j}-\bar{r_{i,j}}\right)}^{2}}{2\sigma_{i,j}^{2}}\right\}$$

Finally we obtain equations (3) and (4);

(3) $$-\frac{\epsilon_{i,j}}{\mathrm{kT}}-\ln\;Z=-\ln\left(\sqrt{2\pi }\sigma_{i,j}\right)-\frac{{\left(r_{i,j}-\bar{r_{i,j}}\right)}^{2}}{2\sigma_{i,j}^{2}}\;$$

(4) $$\frac{\epsilon_{i,j}}{\mathrm{kT}}=\frac{{\left(r_{i,j}-\bar{r_{i,j}}\right)}^{2}}{2\sigma_{i,j}^{2}}-\ln\frac{Z}{\sqrt{2\pi }\sigma_{i,j}}$$

where kT is set so that the acceptance ratio is 0.5. This potential is designed to sample the ensembles which can reproduce the inter-residue average distance statistics. From the simulation, the contact frequency, g(i,j), for each pair of residues is calculated with sampled structures generated using the potential energy function. Then we normalize the residue contact frequencies, g(i,j), in the same range M as follows:

(5) $$D\left(M\right)=\sqrt{\sum_{\left|\mu -v\right|=m}\frac{{\left({\tilde{g}}_{\left|\mu -v\right|\in M}\;-g{\left(\mu ,v\right)}_{\left|\mu -v\right|\in M}\right)}^{2}}{\Sigma_{{}_{\left|\mu -v\right|\in M}}}}$$

(6) $$Q\left(i,j\right)=\frac{g{\left(i,j\right)}_{\left|i-j\right|\in M}\;-\frac{\Sigma_{\left|\mu -v\right|\in M\;}g\left(\mu ,v\right)}{\Sigma_{\left|\mu -v\right|\in M}}}{D\left(M\right)}$$

where *μ* or *v* is the residue number. Finally, the relative contact frequency, F_i_, is obtained by summing the normalized contact frequencies, Q(i,j), from j = 1 to N for each residue i, where N is the protein sequence length:

(7) $$F_{i}=\sum_{j}Q\left(i,j\right)$$

The peaks of the plots of the F_i_- or F-value peaks are thought to be located in the center of many inter-residue contacts, such as a hydrophobic cluster. Therefore, the regions around the peaks are assumed to be important for folding, especially for its initial state. F-value analysis therefore allows us to estimate the location where a folding initiation occurs, except for the termini with their expected extreme flexibility in the simulation: due to the flexibility, the F_i_ values at the terminal residues become unrealistically high, and this value is then considered not to be true [47]. We performed this simulation with 60000 steps 100 times, calculating the average F_i_ value for residue i.

### Analysis of evolutionarily conserved residues

Evolutionarily conserved residues maintain a protein’s function, contribute to its stability, or relate to its structural formation [48-53]. Therefore, conserved residues that have many contacts with other conserved residues in the native structure are thought to be significant indicators of potential folding segments. Based on this idea, we gather the homologous sequences for each study protein with the Basic Local Alignment Search Tool (BLAST) [54] (DB: Uniref100, Threshold: 0.01, Gapped: No) and aligned them with the MUltiple Sequence Comparison by Log-Expectation tool (MUSCLE) [55]. We applied the neighbor-joining method [56] to construct the study protein’s phylogenetic tree for inputting into the Phylogenetic Analysis by Maximum Likelihood software package (PAML) [57]. With PAML, for each site without any gap, we can count the number of residue substitutions by using JTT matrices [58] for the substitution matrix and a Poisson distribution for the substitution model. This procedure allows us to estimate the conservation or substitution of a specific residue during evolution based on branch lengths and bifurcations in a phylogenetic tree. Because only the conservation of hydrophobic residues is taken into account in this study, the hydrophobic residues with more than 99% conservation are regarded as conserved residues, that is, we still regard a residue as conserved, when one of the hydrophobic residues A, M, W, L, F, V, I, and Y has mutated to another one.

We employed the BLAST to identify potential homologous sequences. Only sequences that cover the whole sequence of a study protein were selected as homologous sequences based on the BLAST results. The BLAST search identified at least 100 homologous sequences for each study protein were obtained (see Additional file 1: Table S1).

### Definition of inter-residue contacts

The Shrake-Rupley algorithm [59] was used to define a contact by the decrease in the Solvent Accessible Surface Area (SASA) upon folding. The reduction in the surface area is calculated by the difference between a sidechain’s SASA in the presence of contacts with other residues and that in the absence of contacts. In this study, only heavy atoms are considered, and when the decrease in the SASA reaches 27 Å^2^, the corresponding hydrophobic residue pair is defined as being in contact. This threshold was determined from the decrease in the SASA when two carbon atoms form a contact, namely, 27.27 Å^2^.

### Summary of the experimental results from the literature

**Figure 3:**
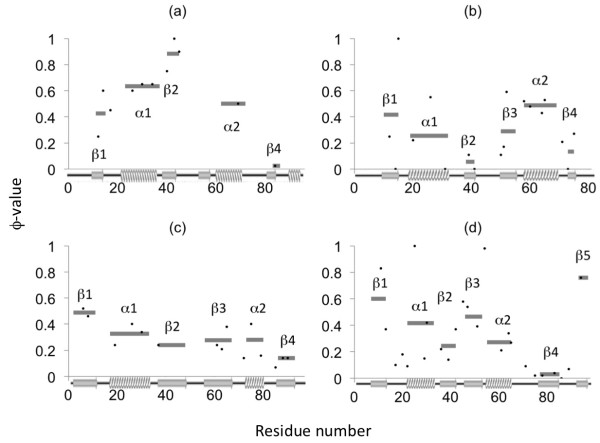
**Experimental Φ values and average values for each secondary structure. (a)** U1A, **(b)** ADA2h, **(c)** S6, and **(d)** mtAcP. Dots denote the experimental Φ values. Gray bars indicate the average Φ value for each secondary structure. Because no Φ values in the 3rd β strand of U1A have been reported, its average value is not shown.

Figure 3 provides a summary of the results reported in the literature from various Φ-value studies which provide information on structured sites in the transition state [60]. We compare the regions predicted by ADM with the location of secondary structures with high Φ values. Averaging the Φ values for each secondary structure is a good way to understand the differences in folding mechanisms among a set of proteins with the same topology and this method has been performed by many researchers (for instance, TNfn3/CD2d1 [61], mt/sso-AcP, [62] mt-AcP/ADA2h, [17,42] wt-S6/permutants [15], and so on). We validated our predictions by comparing them to experimental results interpreted in the same manner. (We need to note that there are a few residues with high Φ values which should be excluded. For example, P54A in mtAcP has a high Φ value of 0.98. However, according to the 3D structure, its side chain seems exposed; thus, its high Φ value seems to be derived from the unique dihedral angles of the proline residue, and we did not treat it as a member of the folding segment).

In this study, a few secondary structures with relatively high Φ values are defined as an experimental folding segment. Even though the resolution is somewhat lower because the folding segment is defined by average Φ values, this approach is still similar in concept to the “folding nucleus” first introduced by Shaknovich and his colleagues [63] as a set of contacts in denatured states that are considered sufficient and necessary for transitioning or molten globule states to occur as observed by a computational technique. The formation of these contacts is rate-limiting step and should be done by its transition state.

Some later studies support the idea of a folding nucleus by means of Φ-value analysis or combining experimental Φ values with computational techniques [64,65]. In other words, the folding segment is thought to be relatively structured compared to other regions from the denatured state to transition state. (This is because high-Φ-value sites correspond to the sites which are energetically stable in the transition state, and we expect that such an energetically stable region forms even in the early stage of folding.) For example, for the wild-type ribosomal protein S6 from *Thermus Thermophilus*, one of the ferredoxin-like folds studied by Haglund et al., [15] one folding nucleus is reported to consist of β1, α1, and β3 (despite the α1 and α2 having very similar average Φ values). However, for the circular permutants prepared by connecting N- and C-termini and disconnecting other loops between neighboring secondary structures, sometimes the folding nucleus noticeably shifts to β1, α2, and β4 [15]. Therefore Haglund et al. [15] found that in S6, there are two competing and overlapping folding nuclei, and the relative magnitude of significance for folding could be perturbed by circular permutation. (In Additional file 1: Figures S3 and S4, we also summarized the results of the ADM and F-value analyses for the circular permutant of S6 whose X-ray crystallographic structure is available. This means there is a guarantee that the native structure is not disordered or structured in other conformations, thereby making its Φ values seem reliable. We obtained similar results as in previous analyses). Ternström et al. [16] also performed an experimental study on U1A spliceosomal protein, which also folds through the α1 formation and its surrounding secondary structures in an early stage. However, Villegas et al. [17] reported that procarboxypeptidase A2, which has the same topology as S6 and U1A, folds through the α2 formation and part of β1/β3. Its folding segments seem to be in the C-terminus (β1, α2, β3) like the S6 permutants (β1, α2, β4 in the S6 wild type), while there are some differences around the β strands. Specifically, there seems to be two tendencies with respect to the location of the folding segments: the N-terminus with α1 and the C-terminus with α2, but it remains difficult to say which β strands contribute to folding. Therefore in this study, we simply chose the β strands that have higher average Φ values compared to the values of the α helix with a lower average Φ value than the other α helix.

## Results

### Folding segments predicted by ADM analysis

**Figure 4:**
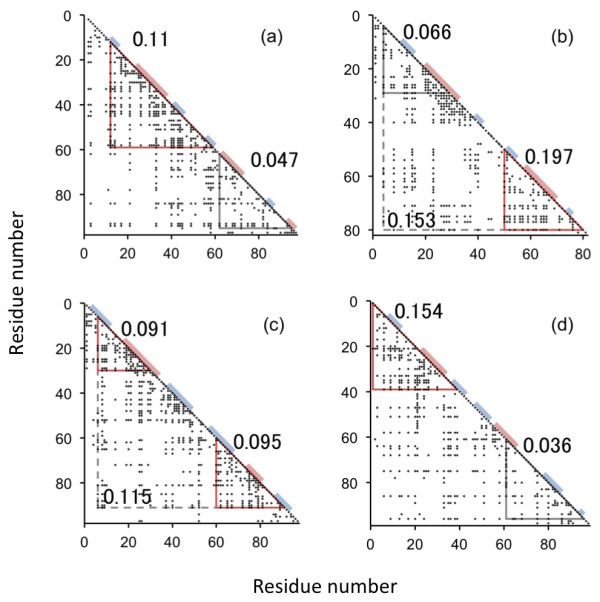
**Results of ADM analyses. (a)** U1A, **(b)** ADA2h, **(c)** S6, and **(d)** mtAcP. The color bars on the diagonal of a predicted contact map indicate the location of secondary structures. The abscissa and ordinate denote residue numbers, and triangles with a solid line in red or black indicate the location of primary or auxiliary compact regions, respectively. A large triangle with a broken line means it is ignored because it covers more than 70% of the entire sequence. η values are shown beside the triangles.

The predicted contact maps and the location of the compact regions, namely, the predicted folding segments are shown in Figure 4 and summarized in Table 1. According to these data, all four proteins have two compact regions, and each region contains one α helix with a couple of β strands.

**Table 1:** Summary of the Average Distance Map (ADM) Analyses

**PDB entry**	**N-termini**	**C-termini**	**η**	**Dominance**
1URN	12	95	0.112^b^	
	12	59	0.110	N
	62	95	0.047	
1O6X	4	29	0.066	
	50	80	0.197	C
1RIS	6	91	0.115^b^	
	6	30	0.091	N
	52	91	0.087^a^	
	60	91	0.095	C
1APS	1	39	0.154	N
	1	47	0.132^a^	
	61	96	0.036	

N or C denote the N- or C-terminal borders of compact regions. The primary compact regions are shown with N or C in the Dominance column. In the η column, compact regions extended by the 85% rule [31] are identified with a superscript a; compact regions ignored due to covering more than 70% of the entire sequence are identified with a superscript b.

**Figure 5:**
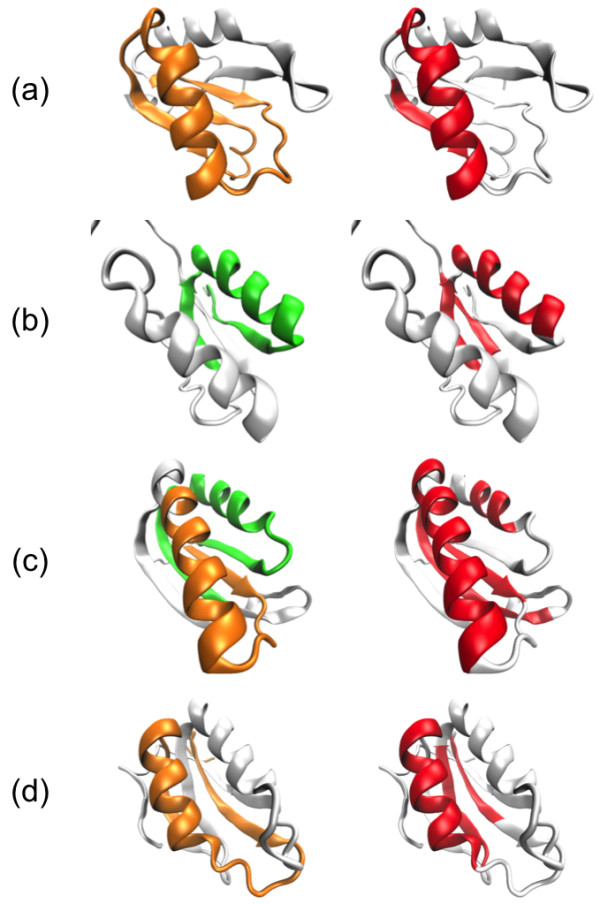
**Comparison of predicted folding segments and experimental folding segments. (a)** U1A, **(b)** ADA2h, **(c)** S6, and **(d)** mtAcP. In the left column, the predicted primary folding segments located at the N- or C-termini are colored orange or green, respectively. In the right column, all the secondary structures with an average Φ value higher than that of the α helix with the lower average Φ value are colored red. However, for S6, the β-strand 3 and α-helix 1 are also colored in red, because their average Φ values are not significantly lower than the averge Φ value of the α helix with the higher value, unlike the case in other proteins.

For U1A spliceosomal protein ([PDB: 1URN]) and muscle-type acylphosphatase 2 [PDB: 1APS], the N-terminal compact region has a higher η value than that of the C-terminus, which suggests that the N-terminal region is stable compared to the C-terminal region during the early stage of folding, while procarboxypeptidase A2 [PDB: 1O6X] shows the opposite trend. As for ribosomal protein S6 [PDB: 1RIS], the two compact regions have similar η values, which is interpreted as meaning that both of these regions play equally important roles in structural formation. It is also notable that except for the case of mtAcP, β3 is always included in the primary folding segments (the predicted region with a higher η value) for each protein.We are interested in comparing the predicted folding segments with the secondary structures whose average Φ values are high. Figure 5a-d show the results. According to these figures, the secondary structures within the predicted folding segments often correspond to the secondary structures with high average Φ values.

In Figure 5, the positions of the secondary structures with higher average Φ values than those of the α helix (taking the lower Φ value of the two α helices) are colored red in the right panel, while in the left panel, the positions of the predicted primary folding segment at the N- or C-terminus are colored yellow or green, respectively. For S6, however, we color both segments in red or yellow/green, because according to the average Φ values and η values of S6 (see Figure 3 and Table 1, respectively), both N-terminal and C-terminal folding segments are equally important in the formation of its 3D structure. Figures 3, 4, and 5 indicate that almost all the important secondary structures for folding, as defined by experimental Φ value results, are included in the folding segments predicted by the ADMs, although β3 in mtAcP, which shows a relatively high Φ value, is not included in the region predicted by the ADM.

### Evolutionary conservation analysis with F-value analysis - Location of predicted hydrophobic clusters

**Figure 6:**
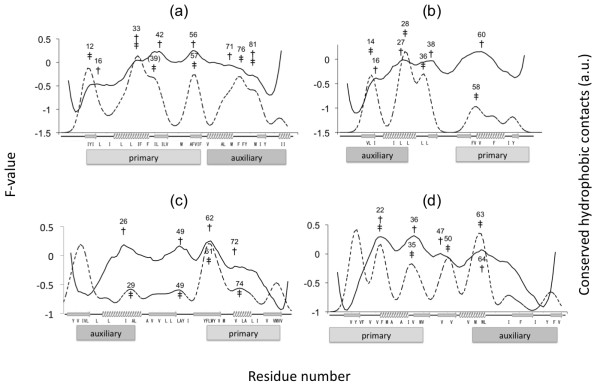
**Results of F-value analyses and the distribution of conserved hydrophobic contacts. (a)** U1A, **(b)** ADA2h, **(c)** S6, and **(d)** mtAcP. F values or the smoothed number of conserved hydrophobic contacts are shown as a solid or broken line, respectively. The ordinate denotes the F value or the number of conserved hydrophobic contacts and the patterns along the abscissa show the location of secondary structures. The conserved amino acid residues and the location of predicted folding segments are also shown below the plot. The F-value peaks that were the focus of this study are marked with single daggers (†), and the number above each dagger denotes the residue number of the respective peak. The smoothed number of conserved hydrophobic contacts is in arbitrary units, and the peak location is shown with a double dagger (‡) like the F-value peaks. Only for U1A, the shoulder is indicated with parentheses.

The solid line in Figure 6 indicates the F-value result, while the broken line shows the smoothed plots of the number of contacts with other conserved hydrophobic residues. (The conserved hydrophobic sites are shown in Additional file 1: Figure S8.) Smoothing was performed with a Gaussian kernel [66]. A conserved hydrophobic contact means that a pair of conserved hydrophobic residues form a contact. The locations of the peaks are indicated by single or double daggers for F-value or conserved hydrophobic contacts, respectively. A number near a dagger indicates the corresponding residue number in a protein. We follow the PDB system concerning the residue number in a protein. The secondary structures and conserved hydrophobic residues are shown below the plot.Except for several sites, most of the conserved hydrophobic residues are distributed somewhat sparsely but uniformly, which implies that it is hard to extract folding segments from only their amino acid sequences and conservation analyses. According to Figure 6, most of the F-value peaks are close to those of the smoothed line within ± 3 residues. This indicates that F-value peaks, which can be mainly regarded as hydrophobic clusters in the initial nucleation stage, also correspond to the region with many conserved hydrophobic contacts, which are important for the formation of a native structure.

Direct comparisons of these regions with high-Φ-value sites are shown in Additional file 1: Figure S9. For high-Φ-value sites, only the sites with a Φ value higher than the average Φ value of the protein are shown along with each site’s residue type and number. The peaks of smoothed conserved hydrophobic contacts are marked by double daggers as in Figure 6. High-Φ-value sites are found to exist near the peaks of the conserved hydrophobic contacts (within ± 3 residues), suggesting that some of these contacts are responsible for structural formation. These high-Φ-value sites are also found to exist near the F-value peaks within ± 3 residues, as shown in Additional file 1: Figure S10.

### Folding segments predicted by ADMs in the homologous proteins of the study proteins

**Figure 7:**
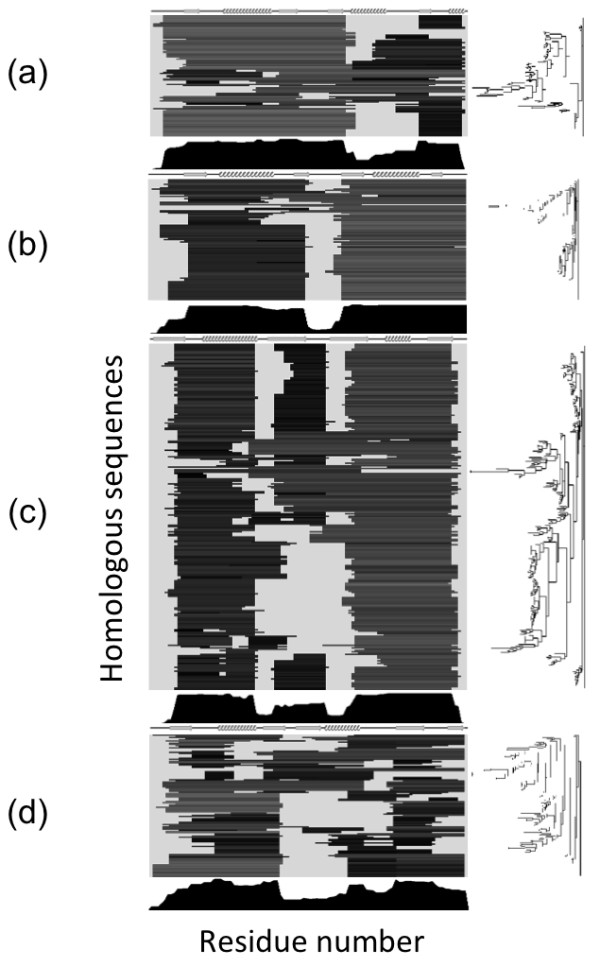
**Results of applying ADM analysis to the homologues of the study proteins. (a)** U1A, **(b)** ADA2h, **(c)** S6, and **(d)** mtAcP. Each line corresponds to one homologue, and the dark gray colored regions are the compact regions predicted by the ADMs. The lighter the color is, the higher its η value. These homologues are sorted by the ADM similarity. On the right side, the tree made from the ADM similarity matrix by means of the neighbor-joining method [56] is shown. The location of the secondary structures or the ratio of being included in ADMs for each site is shown above (by arrows or helices for β strands and α helices) or below the results of the ADM analyses (as a histogram), respectively.

To confirm whether the folding segments are conserved among evolutionarily related proteins, we performed our sequence analysis on the homologues of the four study proteins. The results of applying ADM analyses to these homologues are shown in Figure 7.This figure denotes the respective multiple alignments of the homologues. The location of the predicted folding segments are colored dark gray: the brighter the color, the higher the region’s η value. It can be visually confirmed in Figure 7a-c that there are several bands indicating that for most of the homologues the same regions are predicted.

In Figure 7, we ordered the sequences based on the similarity of the location of the regions predicted by the ADMs. In the right column, the phylogenetic tree based on an ADM similarity matrix and the neighbor-joining method is shown. Another result based on the sequence identity is shown in Additional file 1: Figure S7. It is difficult to determine the relationship between the location of the folding segments and their evolutionary distance (as specified by the calculated sequence identity). However, we can conclude that for U1A, ADA2h, and S6, the folding segments themselves are conserved among their homologues, while those of mtAcP are not.To represent these common folding segments, we calculate the percentage of residues that are members of the predicted folding segments for each site. The results are also shown as a histogram colored black in Figure 7.

In the case of U1A, four secondary structures βαββ at the N-terminus form one strong folding segment for most of the homologues, while the other C-terminal region comprises a weak folding segment. For ADA2h, the C-terminal folding segment βαβ is conservative and strong, and the N-terminal folding segment is conservative but weak.

As for S6, there are many homologues, and they share almost the same folding segments. One segment consists of βαβ at the C-terminus and the other one consists of βα at the N-terminus. The dominance of these two folding segments at the termini often differs among the homologues. It is also notable that for some homologous proteins, the region from β2 to the hairpin-loop comprises the weakest folding segment, which forms a β-hairpin with β3 in the C-terminal folding segment.

Finally, for mtAcP, the folding segments are not conservative among its homologous proteins. However, the locations of the folding segments appear similar to those of S6, ADA2h, and U1A.

## Discussion

The ADM analyses of the four proteins predict two compact regions including one α helix and a couple of β strands for each protein. These regions contain the secondary structures with high average Φ values (Figure 3). Therefore, we consider the predicted compact regions to correspond well to the folding initiation segments as was the case for the other proteins we treated in previous studies, including lysozyme, leghemoglobin, fatty acid binding protein, azurin, and two ancient TIM-barrel proteins [33-36]. According to the η values, mtAcP and U1A have the primary predicted folding initiation segment at the N-terminus, whereas ADA2h has one at the C-terminus. On the other hand, the two folding segments of S6 have similar η values (see Figure 4). Figure 5 shows good agreement between the ADM predictions and the experimental results; however, the resolution of this analysis is too low to predict the folding mechanisms.

By means of F-value analysis, we increased the resolution of the prediction made by ADM, allowing us to identify the regions that would form some hydrophobic clusters. According to Figure 6, almost all the peaks are located on the secondary structures or their edges, and the highest peak is located in the primary folding segment for each protein. For example, U1A has the primary folding segment at the N terminus from β1 to β3, and its highest F-value peak is located in β3. In addition, a peak of F values is located at a peak of the smoothed line of the conserved hydrophobic contacts within ± 3 residues, except for the broad peak observed at the C-terminus of ADA2h, which contains several minor peaks. These conserved contacts are thought to play important roles in the structural formation or stabilization of U1A’s native structure [48-53]. Taking these facts into account, the conserved hydrophobic residues near the F-value peaks are considered to be significant for the folding initiation. The basis for predicting the folding mechanisms from only sequence information is the fact that the regions predicted by ADM analysis contain the high-Φ-value residues measured by experiments [15-17,42] and that the F-value analysis reflects the conserved hydrophobic contacts. Let us now make inferences regarding the folding mechanisms for proteins based on the results of the ADMs and F-value analyses.

### U1A spliceosomal protein; U1A

**Figure 8:**
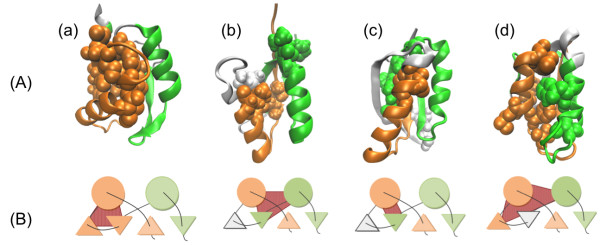
**Hydrophobic interactions observed in the ferredoxin-like fold proteins. (A)** Representation of internal conserved hydrophobic contacts. N- and C-terminal compact regions are colored yellow and green, respectively. The conserved hydrophobic residues near the F-value peaks have a space-filling representation. **(B)** Illustrations of important interactions among the secondary structures discussed in the current study. α helices are shown as circles, and the β strands are shown as triangles. The N- or C-terminal compact regions are colored in yellow or green, respectively, as in **(A)**. The important interactions are indicated in red.

As shown in Figure 6a, the primary compact region of U1A covers β1, α1, β2, and β3, and each region of α1, β2, and β3 contains just one F-value peak. The auxiliary compact region of U1A ranges from α2 to α3. Because the auxiliary compact region has a lower η value, it is thought to participate in the structural formation after the primary compact region has been formed. Since Ternström et al. [16] suggest that the region from β1 to β3 is more structured compared to α2 and β4 [16,67], we find that our results agree well with their experimental Φ-value analysis. Figure 8a presents the packing formed by conserved hydrophobic residues near the F-value peaks. The residues that contribute to the hydrophobic packing are represented in the CPK model in this figure. The regions colored yellow or green correspond to the predicted N- or C-terminal compact regions, respectively.

### Procarboxypeptidase A2; ADA2h

The auxiliary compact region of ADA2h at the N-terminus has two secondary structures, β1 and α1, whereas the primary compact region at the C-terminus has three secondary structures, β3, α2, and β4. The former has a high F-value peak around α1, indicating that α1 is the center of folding within the auxiliary compact region. On the other hand, the primary compact region has the highest and broadest peak from β3 to α2. Therefore, we can predict that after β3 and α2 form a folding segment, β1 and α1, pack with this segment and stabilize it.

This prediction can also be validated by Figure 3b. Villegas et al. [17] state that the folding segment in this protein consists of α2 and its surrounding β strands. This agrees with our result. However, we could not confirm any packing between the conserved hydrophobic residues near the F-value peaks in Figure 8b within the primary compact region in the native structure as observed in U1A. This is because the region with the largest broad F-value peak in the C-terminal region seems to have only a few conserved hydrophobic residues as indicated by the smoothed plot of the conserved contacts which shows several minor peaks here. In this case, the resolution of the F-value line is too low to detect the residues important for folding.

### Ribosomal protein S6; S6

The relative auxiliary folding segment of S6 at the N-terminus contains β1 and α1, while the primary folding segment at the C-terminus contains β3, α2, and β4. The η values are quite similar, so we cannot say which region folds more dominantly. S6 has two significant F-value peaks within the predicted folding segments: one is around α1 at the N-terminus, and the other is around β3 at the C-terminus. Lindberg et al. [67] suggest that the primary folding segment consists of β1, α1, and β3 in the early folding stage, and our results reflect this. Figure 8c shows the residue packing near the F-value peaks inside the predicted folding segments.

It is also notable that in the case of S6, there is a highly frustrated region between the C-terminal unstructured coil and the β sheet based on the structure of S6 [68]. However, the corresponding C-terminal region does not have any specific contact with other regions in the ADM. This is confirmed by its NMR structure ([PDB: 2KJV]). At least as far as concerning the ADM result and the NMR structure, the frustration between the C-terminal unstructured coil and the β sheet does not seem strong.

### Muscle-type acylphosphatase 2; mtAcP

The primary folding segment of this protein, which has a significantly higher η value than the other folding segment, is located at the N-terminus. This result is the same as the result from Selvaraj et al. [69] who suggested the existence of a hydrophobic cluster surrounding α1 based on the distribution of contacts. The primary segment contains β1, α1 and β2, whereas the auxiliary segment contains α2, β4, and β5. There are four F-value peaks near α1, β2, β3, and α2; three of them are located in the predicted folding segments, but the peak near β3 is not. (In fact, β3 belongs to the primary folding segment and seems to play a critical role in the folding in other proteins.) Therefore we propose that after α1 and β2 play a role in early structural formation, α2 participates in the last structural formation, followed by contributions from β3. This scenario does not seem to fit the results of the average Φ-value analysis [42], which indicate that β3 plays a more important role than β2 (see Figure 2d).

According to Parrini et al. [70], when β3 is forced to join the folding process by a disulfide bond between β1 and β3, the folding rate is improved dramatically. This result suggests that the participation of β3 in the folding process is rate limiting and may reflect the present findings. For this reason, we do not consider the two analyses to conflict with each other. The inter-segment packing between the conserved residues is represented in Figure 8d.

Meanwhile, it should be noted that in their analysis, Chiti et al. [42] ignore the highest Φ value of the 23rd residue located in α1 and then compare the result with that of ADA2h. In this case, the average Φ value of α2 is higher than that of α1, indicating that the more structured secondary structure in its transition state is α2, the same as for ADA2h. There are also several studies that suggest that the α2 in mtAcP is more important than α1 [71-74]. Interestingly, one of the studies refers to the large effect on α1 induced by point-mutation. Taddei et al. [71] consider the Φ values of α1 to be unreliable because, according to their experiment, inducing a point-mutation on α1 makes mtAcP form amyloid fibrils. Thus, interpreting the folding segment of mtAcP is difficult.

**Figure 9:**
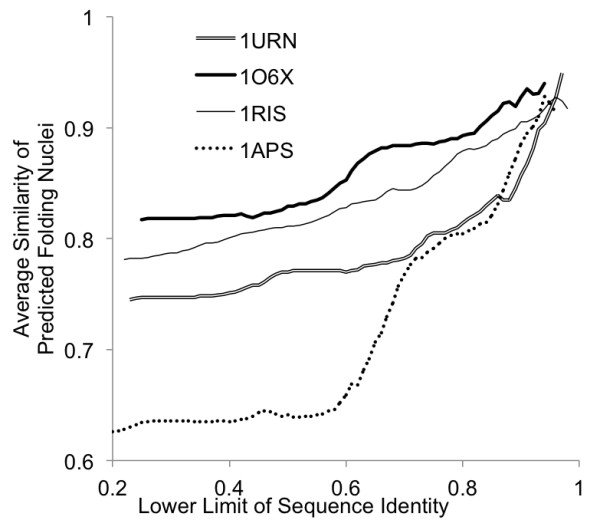
**The relationship between the similarity of the predicted folding segments and sequence identity.** The abscissa denotes the lower limit of sequence identity and the ordinate denotes the average similarity of the predicted folding segments. The double line, solid line, thick line, and dotted line correspond to U1A, ADA2h, S6, and mtAcP, respectively.

Our previous simplified Go-model simulations reveal that the interactions between the folding segments in the present definition are significant in the formation of transition state ensembles [75].According to the discussion above, the conserved hydrophobic residues among homologues are distributed near F-value peaks (Figure 6), and they seem to be involved in folding. Yet, as we mentioned in the introduction section, there are some studies that have shown that several sequences that fold into the same 3D structure have the same folding process, while other studies have shown that two evolutionarily related sequences with the same 3D structure could have different folding processes. In this sense, whether all the homologues of our study proteins really have the same folding segments or not would be an interesting question.In the present study, we aim to analyze the conservation of the predicted folding segments in the homologues of a protein by applying ADM analysis to them. The predicted folding segments are highly conserved among the highly homologous proteins (with roughly 50% sequence identity on average) except for mtAcP and its homologous proteins, as shown in Figures 7 and 9. In Figure 7, the conservation of the predicted folding segments is summarized as a histogram below each result. As seen in this figure, the histogram of mtAcP is uneven compared to the other histograms. Figure 9 depicts this property from another aspect.

The abscissa in Figure 9 represents the lower limit of the sequence identity for calculating the average ADM similarity, that is, an average ADM similarity value is calculated using homologues with a sequence identity of more than a lower limit, while the ordinate denotes the average ADM similarity. The doubled line, solid line, thick line, and dotted line correspond to U1A, ADA2h, S6, and mtAcP, respectively. While the other three proteins maintain their folding segment similarity of more than 75% even when the sequence identity decreases; only mtAcP loses similarity down to 62%. This result suggests a diversity of folding processes in mtAcP compared to those in the other proteins, especially when sequence identity is low. On the other hand, as we mentioned in the results section, the relationship between ADM similarity and sequence identity is not parallel (see Additional file 1: Figure S7). This is an unexpected result: we expected that the more similar the sequence identity is, the more similar the protein folding segments are. Yet the present results suggest that a property related to folding segments is conserved more than sequence identity.

Summarizing the discussion above, there are mainly two situations. One of them clearly comprises a main large folding segment around α1, like in U1A. The other situation comprises complex folding segments in which, one of the α helices and its surrounding β strands play a key role at first, immediately followed by the other helix and its surrounding strands, as in ADA2h or S6. The homologous proteins of mtAcP have either property: some of them have folding segments similar to those of U1A proteins, and some others have folding segments similar to those of S6 or ADA2h proteins. This implies that mtAcP and its homologous proteins do not seem to have any common or rigid folding segments.

## Conclusions

The secondary structures that are thought to play important roles in folding as revealed by their average Φ values correspond to the folding segments predicted by ADM analyses at least for the proteins treated in this study, as was the case in our previous studies [33-36,47,76]. There are two predicted folding segments at the termini of each protein; however, which segment is primary is completely determined on a case-by-case basis. This tendency was also in good agreement with the experimental results for the present four study proteins. Some of the conserved hydrophobic contacts considered to play important roles in structural formation [49,53] are located near the F-value peaks. Therefore, we can predict the folding mechanisms by extracting the conserved hydrophobic residues near them. For the four proteins we studied above, we conclude that we succeeded in predicting their folding mechanisms correctly from only their sequences.

According to the ADM results of the homologues, their folding segments seem to be conserved, especially when the sequence identity is above 80%. Below this level, only mtAcP represents a diversity of folding segments, whereas the other three proteins show high conservations.

Our findings suggest that it should be possible to predict the folding mechanisms or properties of many other kinds of proteins from only the amino acid sequences by means of our ADM analysis and F-value analysis.

## Abbreviations

ADM: Average Distance Map; BLAST: Basic Local Alignment Search Tool; SASA: Solvent Accessible Surface Area; PDB: Protein Data Bank; MUSCLE: MUltiple Sequence Comparison by Log Expectation; PAML: Phylogenetic Analysis with Maximum Likelihood; JTT: Jones Taylor Thornton.

## Competing interests

The authors declare that they have no competing interests.

## Authors’ contributions

TK conceived and designed the basis of this study. MM performed all the calculations and data analysis. TK and MM wrote the manuscript. Both authors read and approved the final manuscript.

## Supplementary Material

Additional file 1 Details of the ADM analysis and optional results are provided. Click here for file
