# Supplementary material for: Sequence analysis on the information of folding initiation segments in ferredoxin-like fold proteins
Source: BMC Struct Biol. 2014 May 23;14:15. doi: 10.1186/1472-6807-14-15 (PMC4055915; doi:10.1186/1472-6807-14-15)
Supplement: Additional file 1 — Details of the ADM analysis and optional results are provided. [file 1472-6807-14-15-S1.pdf]

Supporting Information

Average Distance Map analysis

Construction of a predicted contact map

We plot a pair of amino acids whose average distance is shorter than a specified cutoff. The average distance for each pair of residues is calculated with 42 representative globular proteins. The specified cutoff is set by two criteria. First, the plot density of whole area in a predicted contact map should be similar to the actual contact maps. If an actual contact map whose cutoff is 10 Å, the plot density would be 8.39 divided by its sequence length. Second, the smaller the M value is, the higher plot density the corresponding area should have. To meet these criteria, the plot-density gradient is set to make the density, d(M), obey d(M) = 8.39/M.

Extracting compact regions from a predicted contact map

After the predicted contact map is made with the specified cutoff set by the two criteria described above, all the compact regions are extracted. If there is a structural unit, it will appear as a high-plot-density area in the predicted contact map. By calculating plot-density differences between the triangular and trapezoidal areas

separated by a vertical line for each residue site (see Figure S2), we define the N-terminal borders of the folding units at the maxima of the plot-density differences (see Figure S1 and S2 for more detail).

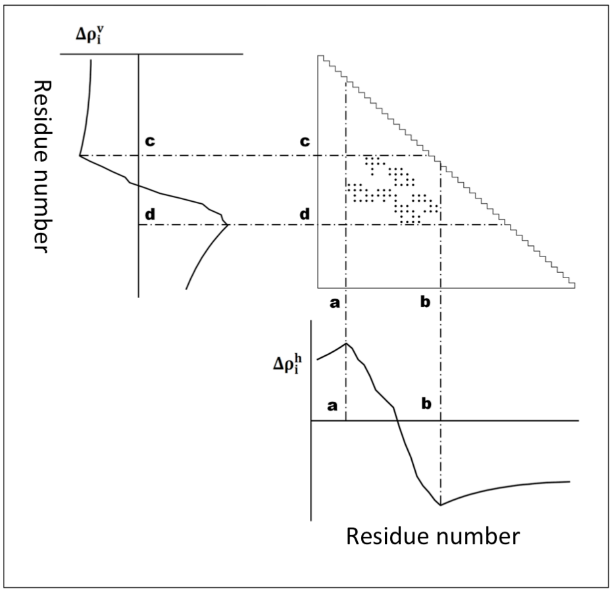

Figure S1: Example of scanning plot-density differences to determine the borders of structural units. **a**, located at the peak of the horizontal scanning plot, denotes the N-terminus of some structural unit, while **d**, located at the peak of the vertical scanning plot, denotes the C-terminus. The η value, the strength of the corresponding structural unit, is calculated by summing the plot-density differences between these two peaks.

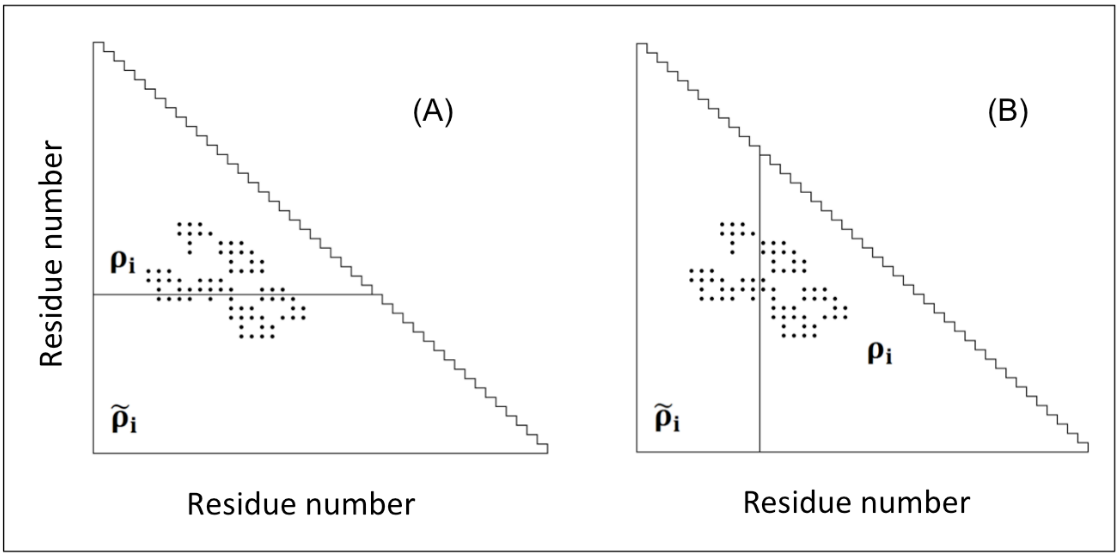

Figure S2 (A) Details of plot-density difference for a vertical scanning plot. ρ_i denotes the plot density of a triangle, and $\tilde{\rho}_i$ denotes the plot density of a trapezoid area separated by a horizontal line. The borders of structural units are determined where $\rho_i - \tilde{\rho}_i$ becomes a local maximum. (B) Corresponding details for the horizontal scanning plot.

We can also define the C-terminal borders by the same procedure using a horizontal line instead. The sum of the two plot-density differences is called the η value, and we take this value as indicating the importance of the predicted folding segment. Only the compact regions which do not overlap the compact regions with higher η values are extracted as predicted compact regions, i.e., predicted folding segments.

Analyses on the circular permutant

The ϕ values of the circular permutant P54-55 of S6 are shown in Table S2.[15] Residue numbers are renumbered starting from 1 at the N-terminus. The structure is

available in PDB with the ID, [PDB: 2KJW]. The ADM and F-value analyses for this protein are shown in Figures S3 and S4, respectively. As is the case for the other Ferredoxin-like proteins, this protein also has two predicted folding segments. According to the ϕ values and the F-value analysis, all the high ϕ values and the highest F value are located in the N-terminal folding segment, thus the primary folding segment seems to be the N-terminal one. From the ADM analysis, the N-terminal folding segment has a similar η-value to the C-terminal segment, and it is somewhat difficult to determine which is the primary folding segment based on the ADM analysis itself.

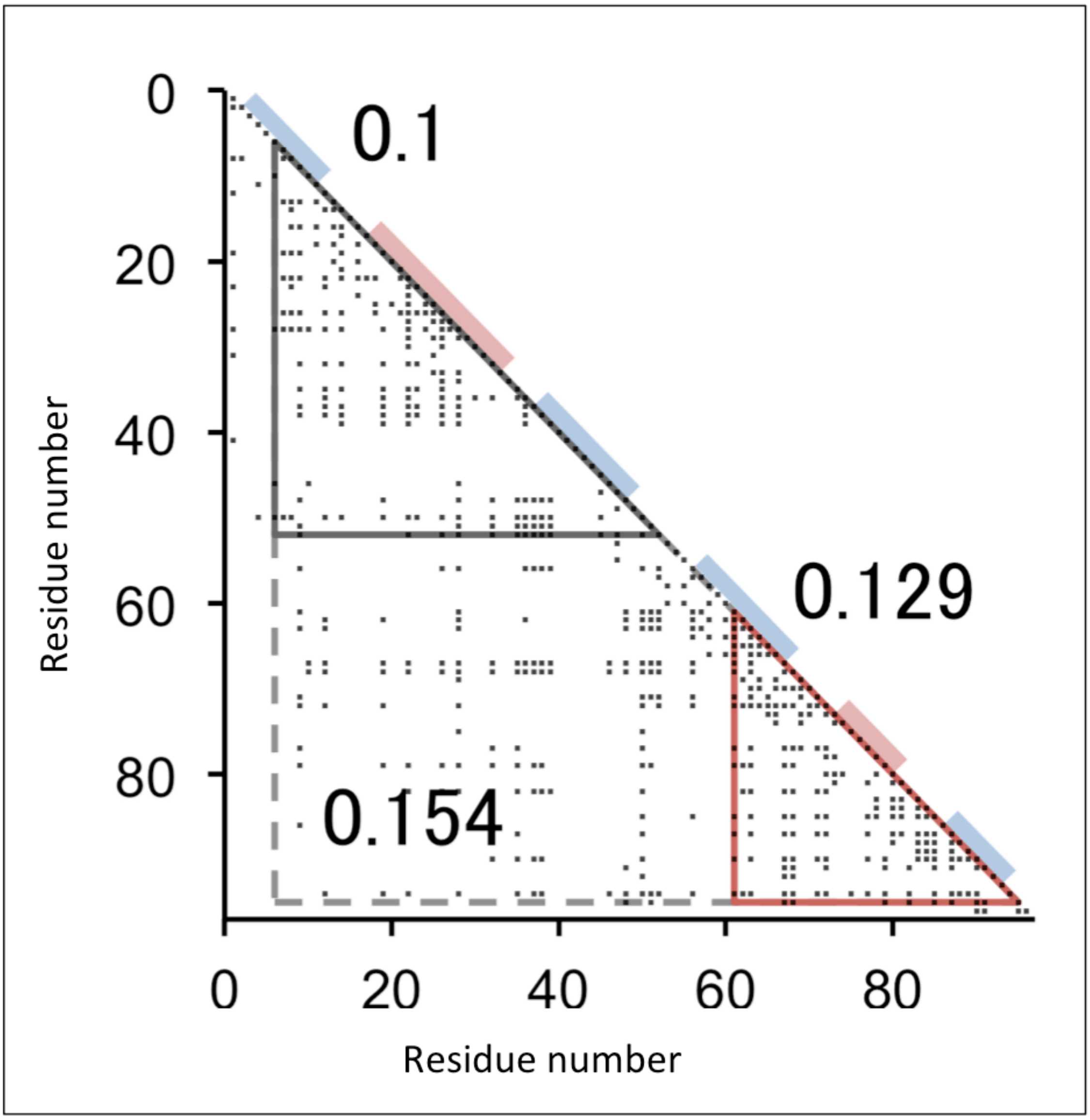

Figure S3: Result of ADM analysis for S6 circular permutant (2KJW) The color bars on the diagonal of a predicted contact map indicate the location of secondary structures. The abscissa and ordinate denote residue numbers, and triangles with a solid line in red or black indicate the location of primary or auxiliary compact regions, respectively. A large triangle with a broken line means it is ignored because it covers more than 70% of the entire sequence. η values are shown beside the triangles.

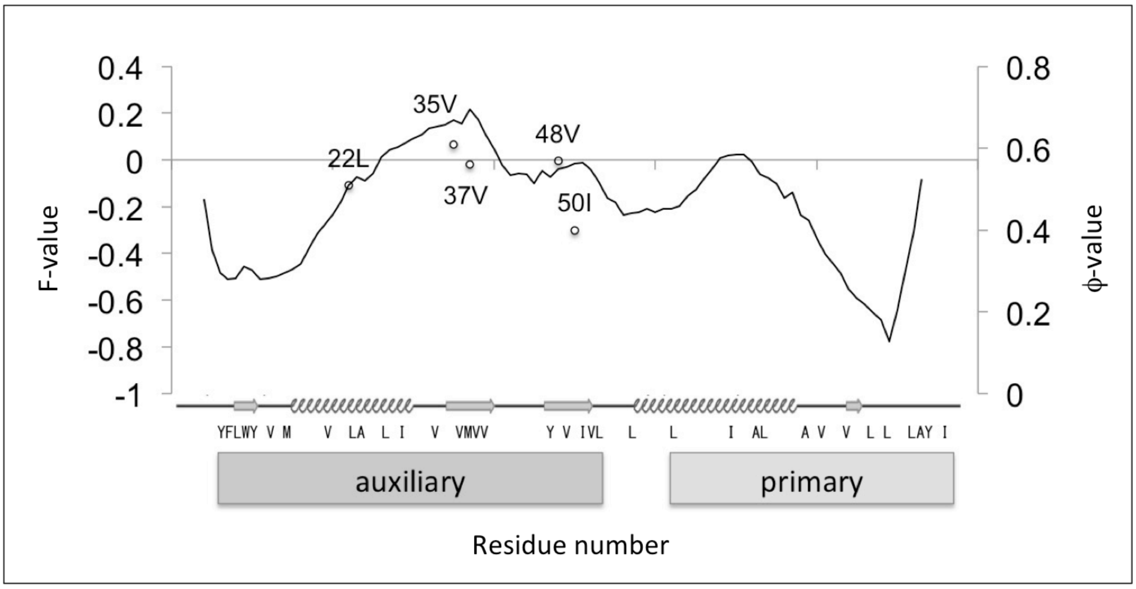

Figure S4: Results of F-value analysis and high ϕ-value residues for S6 circular permutant (2KJW) F values and residues with high ϕ values are shown. The ordinate denotes the F value and the patterns along the abscissa show the location of secondary structures. The conserved amino acid residues and the location of predicted folding segments are also shown below the plot.

The comparisons between the ADMs and the actual contact maps of the Ferredoxin-like proteins

We present the ADMs and the actual contact maps of the Ferredoxin-like proteins in Figure S5. The figures show that the predicted folding segments correspond to the assigned compact regions in the actual contact maps. Due to the properties of ADM, a smaller region is predicted compared to a region assigned by an actual contact map, that is, ADM predicts a folding nucleus.

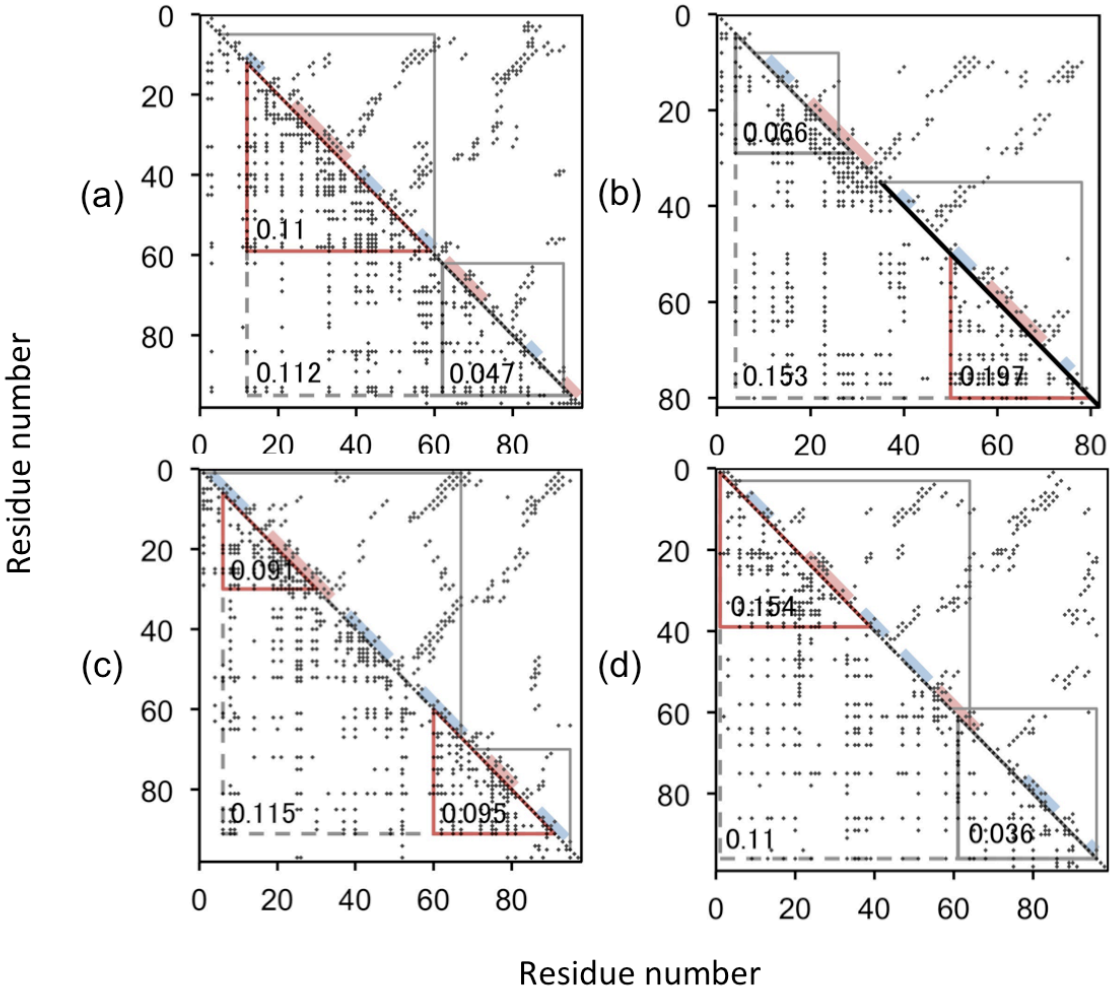

Figure S5: Comparison of ADM and an actual contact map

(a) U1A, (b) ADA2h, (c) S6, and (d) mtAcP. The color bars on the diagonal of the contact maps indicate the location of secondary structures. The lower left contact map is already shown in Figure 4. The upper right contact map is the actual contact map using the definition of contact in this study. The abscissa and ordinate denote residue numbers, and triangles with a solid line in red or black in the lower left contact map indicate the location of primary or auxiliary compact regions, respectively (η values are shown inside the triangles). A large triangle with a broken line in the lower left contact map means it is ignored because it covers more than 70% of the entire sequence. Compact regions defined from the actual contact map are also shown with a gray solid line. The region corresponding to the primary predicted compact region is often extended.

Supporting Information: Illustrations and figures

Figure S6: Comparison of the ADM results with ϕ values. (a) U1A, (b) ADA2h, (c) S6, and (d) mtAcP. In addition to Figure 3, this figure also shows the folding segments defined by ADM analyses, indicated with semi-transparent black squares. The ordinate axis on the right side indicates the η value, while on the left side, the ϕ value is shown. The abscissa axis indicates the residue number. According to these results, primary folding segments often are located where high ϕ-value residues are distributed.

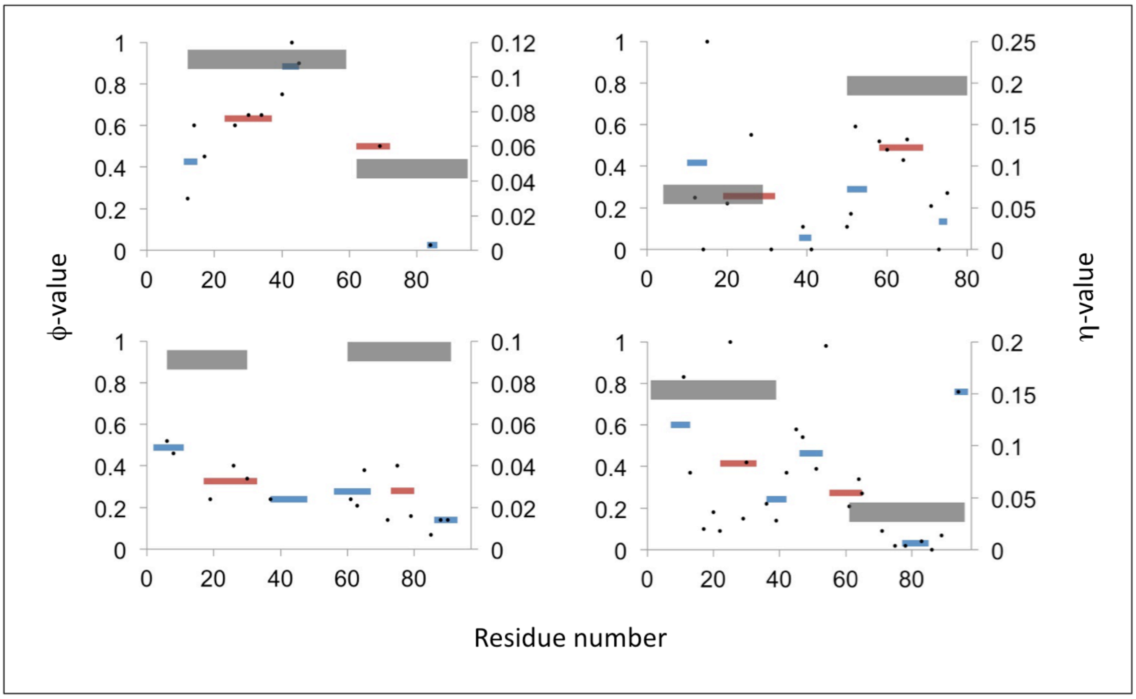

Figure S7: Results of applying ADM analysis to the homologues of study proteins.

(a) U1A, (b) ADA2h, (c) S6, and (d) mtAcP. Only the order of the homologues is different from Figure 7. These homologues are sorted by their sequence identities. On the right side, the phylogenetic tree is shown.

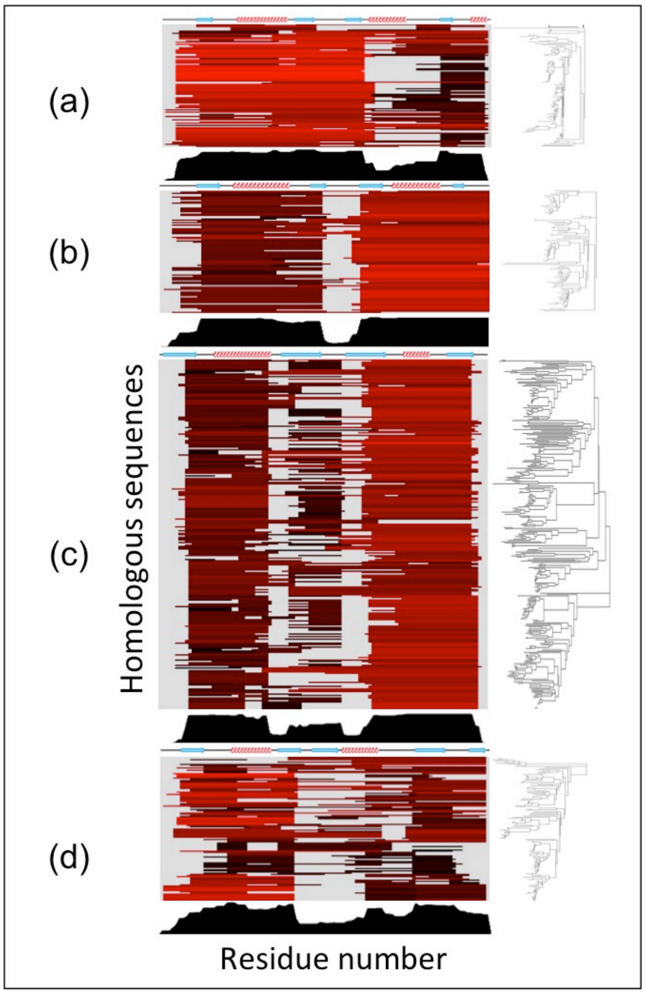

Figure S8: The distribution of conserved hydrophobic residues. The conservation of each residue calculated by the procedure in "Analyses of evolutionarily conserved residues" in the Methods section is shown. (a) U1A, (b) ADA2h, (c) S6, and d) mtAcP. A gray bar corresponds to the conserved hydrophobic residues, and a white bar corresponds to hydrophilic or diverse residues. Arrows and helices along the abscissa denote the β-strands and α-helices.

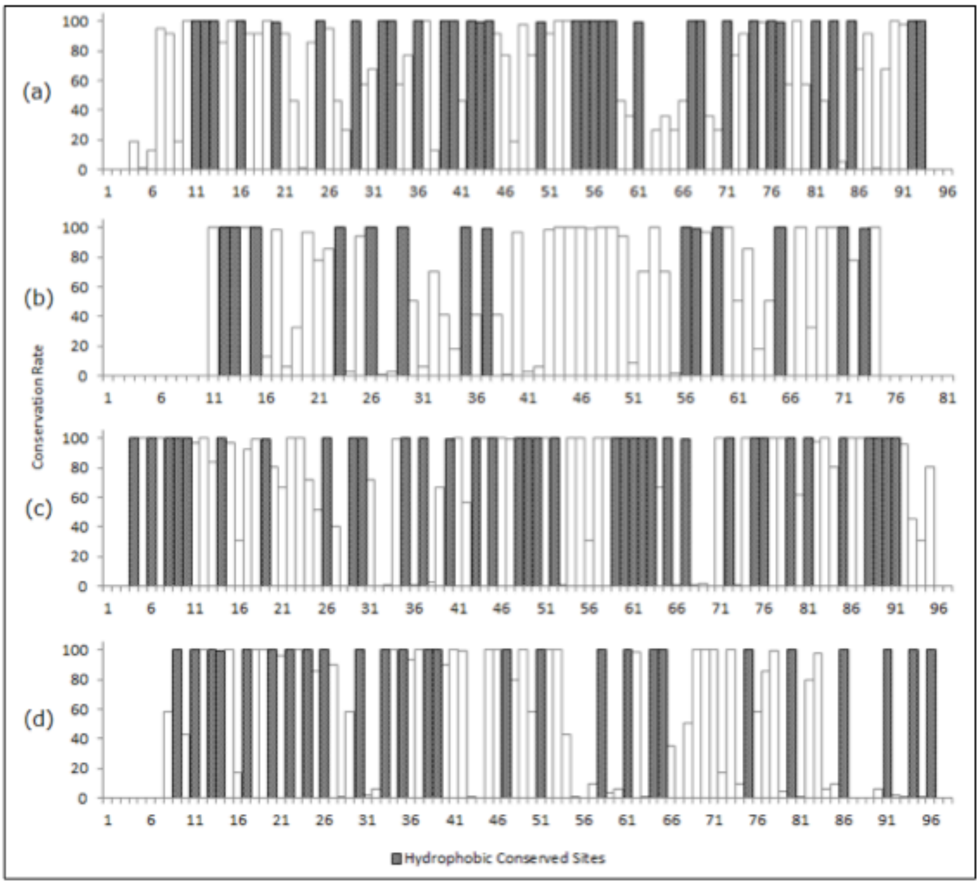

Figure S9: Comparison of the location of conserved hydrophobic contacts with high-ϕ-value sites. Residues with high ϕ value (above the average ϕ value within each protein) are shown with open circles. Also shown are the smoothed number of conserved hydrophobic contacts. (a) U1A, (b) ADA2h, (c) S6, and (d) mtAcP. Except for the residues around the 45th residue in U1A, 52th in ADA2h and the 65th in S6, it can be shown that relatively-high-ϕ-value residues are located near the peaks of smoothed conserved hydrophobic contacts within ± 3 residues. The parentheses of the 39th residue denotes it is not a peak but there is a relatively high number of conserved hydrophobic contacts according to the smoothed number of the contacts. Arrows and helices along the abscissa denote the β-strands and α-helices.

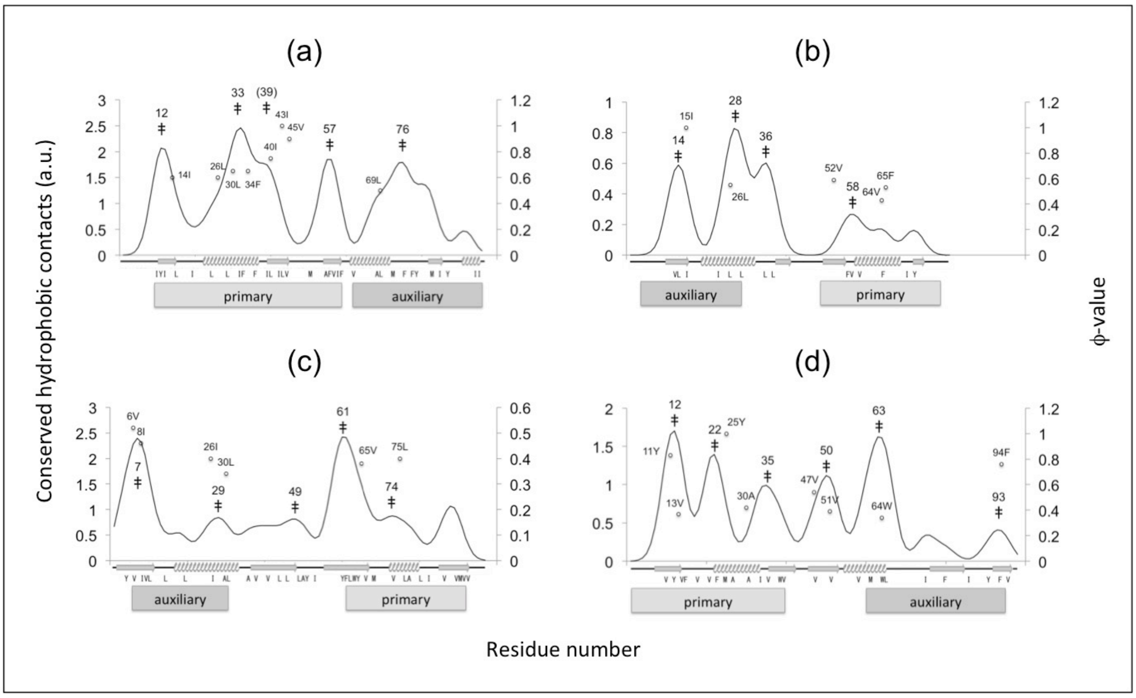

Figure S10: Comparison of the location of F value peaks with high-ϕ-value sites. The high ϕ value residues (above the average ϕ value within each protein) and F value peaks are shown with open circles and a solid line, respectively. (a) U1A, (b) ADA2h, (c) S6 and (d) mtAcP. Except for the residues underlined like the residues near the C-terminus of ADA2h, it can be confirmed that relatively-high-ϕ-value residues are located near the F-value peaks within ±3 residues. Arrows and helices along the abscissa denote the β-strands and α-helices.

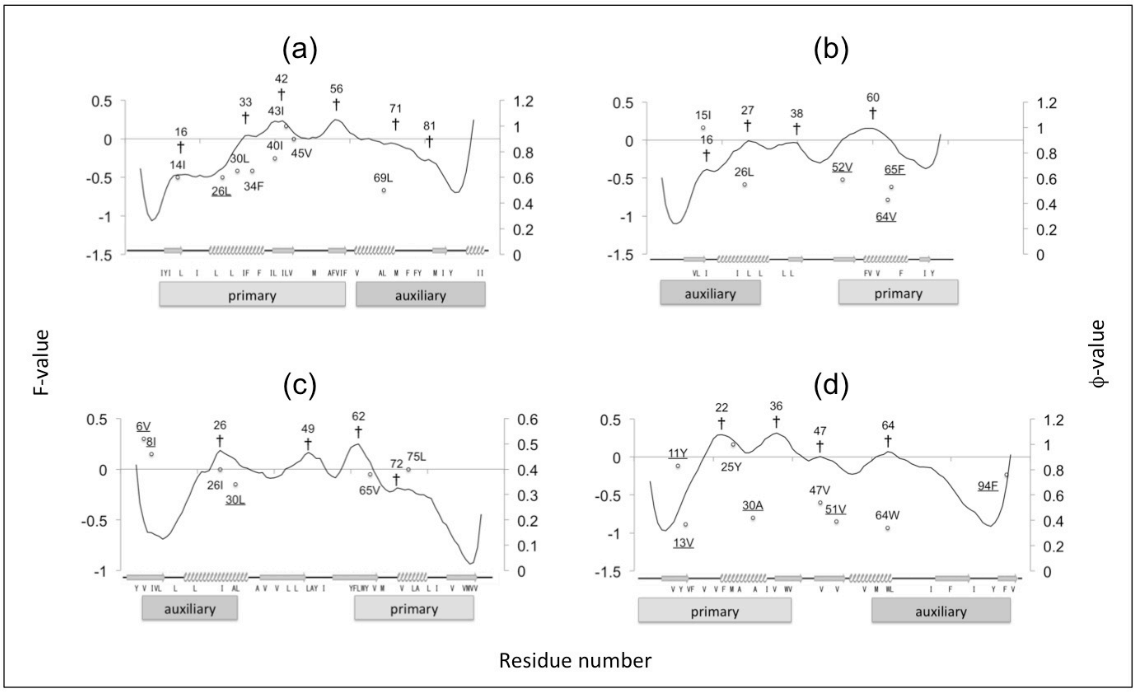

Supporting Information: Tables and captions

Table S1: The Uniprot IDs of the homologues of our study proteins

	Uniprot ID									
	UPI00004BEF8E	A4VEH0	B4JE85	B4LUK6	B5G0Y4	C4WXP9	F1RC85	G1MJ31	P14621	Q5DFQ8
	UPI0000512C7C	A7RUU0	B4JFL9	B4LX48	B5G0Y5	D4A6X4	F3YAZ6	G1NJC9	P24540	Q5EBE1
	UPI000051A176	B0WQ81	B4JPK3	B4M394	B5G0Y6	D6X171	F4X587	G1P9X8	P35744	Q7Q4A5
	UPI00005A2295	B3LWW5	B4JUR5	B4N4L3	B5G0Y7	E0VK32	F6SQ68	G1RCZ6	P35745	Q9GPM0
	UPI0000E239D7	B3M373	B4JVI0	B4NL34	B5G0Y8	E0VW38	F6WNY7	G1SYS6	P41500	Q9VF36
	UPI00015B4DD9	B3MD06	B4JWQ4	B4NLM3	B5G0Z0	E1ZBA5	F7AJT1	P00818	P56375	Q9VHX3
1APS	UPI00016E2EC9	B3MLL5	B4JY70	B4P089	B5G0Z3	E1ZUY9	F7DGK4	P00819	Q16Z05	Q9VMR9
	UPI000194BEE5	B3N4V0	B4K2X3	B4PQY1	B5XBL2	E2AI69	F7EHC1	P00820	Q29GR0	
	UPI0001CBA571	B3NAB3	B4KBY9	B4PRK9	B7PMU2	E2QZI2	F7GV91	P00821	Q29M10	
	UPI0001D5D732	B3NRD9	B4KI43	B4QEX3	B7Q5G1	E3TED5	F7IL81	P07031	Q4RLW7	
	UPI00021A67AA	B3NYU5	B4KT78	B5DI60	B9PKZ0	E9GS27	G1KM45	P07032	Q4TCC0	
	UPI00021A73B6	B3P3V2	B4L2W9	B5DLQ6	C3YXB3	E9IA53	G1KT64	P07033	Q4V6I0	
	UPI0002233D30	B4HLW7	B4LRB0	B5DYC8	C4WSX7	F0VGX1	G1LCT3	P14620	Q4VAF0	
	UPI000012106C	UPI0001CE0AB4	UPI00020E4F2F	A0EZX9	E3TDL7	F6ZBT8	G1PSA8	Q8AXN4		
	UPI00001CF460	UPI0001CE0AB5	UPI00020E4F30	B5X8K9	E3TGR3	F7BPS7	G1RQH3	Q8AXN5		
	UPI000048D2EE	UPI0001CE0AB6	UPI00020F74AF	B5X8N0	E9PYY7	F7E0P9	G1RQJ7	Q8UUK1		
	UPI00005A2B2B	UPI0001D557E3	UPI00020F74B0	B9V313	F1MUG4	F7E8Y9	G1SS65			
	UPI0000E2175B	UPI0001D5EDD1	UPI00021072F0	C1BHH4	F1N6R3	F7E9F3	G1TYU0			
	UPI0000E7F78E	UPI0001D5EDD3	UPI0002107781	C1BLM9	F1NWB2	F7H981	P00731			
1O6X	UPI0000EBC86D	UPI0001D5EDD5	UPI0002107783	C1BZD6	F1QL16	F8WF19	P09954			
	UPI000155E004	UPI0001DE9B2B	UPI0002107784	C3KGU4	F6PGP0	G1LFW6	Q4QXK9			
	UPI000155E470	UPI0001F9A337	UPI000214B238	C3KHL4	F6T293	G1LFX7	Q4VBJ2			
	UPI00016DFA05	UPI000203B462	UPI0002233B53	C3ZNA3	F6T8K2	G1LG84	Q504N0			
	UPI00016EA36C	UPI000203B463	UPI0002233B54	D3ZHS5	F6WX16	G1MVU5	Q5U2W3			
	UPI000194E075	UPI00020AB663	UPI0002233B59	E2RJJ9	F6Y7X1	G1MVW5	Q6IE16			
	UPI000194E076	UPI00020AB665	UPI000223E60E	E2RJK2	F6YU14	G1PS90	Q6P8K8			
	UPI000194E077	UPI00020E4F10	UPI0002256441	E2RJK9	F6ZBR6	G1PS98	Q7TPZ8			
	UPI0000512569	UPI0001D615D4	A8XIX4	B7FJ52	C4WV94	E1FN17	F7E984	Q21322	Q5XHF0	
	UPI00005A15D1	UPI0001E6B732	A9SK98	B9ELD1	C6SZ90	E2C3U3	F7FGA0	Q21323	Q6C824	
	UPI00015B55F5	UPI00020399D6	A9VCS6	B9GSB4	C6TMN2	E3NK70	F7GG89	Q29J33	Q6GQE6	
	UPI00016E4DD3	UPI00021A3DEC	B0DFM6	B9SAT1	C8VFF2	E3NK71	F7GII6	Q39244	Q6P2T1	
	UPI00016E4DD5	UPI00021A816C	B0XIL2	C0H5A4	D3BQD0	E3TDA2	F7HA81	Q3HR40	Q6PBZ6	
	UPI00016E963E	UPI00022360F8	B2R802	C0HB90	D3PJS0	E3XD41	F7I9G0	Q41498	Q7PYY3	
1URN	UPI00017B4E30	UPI00022578FD	B3LBN1	C1BK08	D5GL25	E4Y552	G0PEW9	Q4SPK8	Q7RD14	
	UPI000180CED2	A0DUA6	A5JZ61	B3MRQ8	C1BQ46	D6R728	E9HAA5	G1K8L0	Q4UD23	Q7ZXI5
	UPI000194C02C	A0DWP2	A7AN41	B4NCD2	C1C2H3	D6WG78	F1KZB9	P43332	Q4Y5S2	Q8I2G9
	UPI0001BE8109	A0E3G6	A7RKX3	B4PZS0	C1C334	D7LGP5	F4P516	Q0CFW0	Q4YVM0	Q9CXX7
	UPI0001CA67D9	A0E4Z7	A8PZ57	B5BTZ8	C1C3U7	D8RBP4	F6Y8Y8	Q16H40	Q5BL54	
	UPI0001CBB2E2	A1CE39	A8XIX3	B5XCU4	C3Y3M6	E0W1I4	F6YE01	Q1WCL9	Q5XGC3	

	Uniprot ID										
	UPI0001AF5883	A8LQQ5	B6B4T7	C3BB81	D0CY22	D6GT01	E2CQ77	F3QRS2	Q0FG68	Q65CP3	
	UPI0001DD0768	A9E753	B6GDU0	C3CB91	D0D1W9	D6HKP1	E3E2K6	F3SCN4	Q0FTM4	Q67J48	
	UPI000225B802	A9H1N0	B6IN79	C3P3E5	D0WHE3	D6Y1K6	E3H339	F3ZYJ9	Q0G6L7	Q6ABW7	
	UPI000225E7B2	A9IRU5	B6R7Q0	C4FB00	D1PY71	D6ZB09	E4LXE2	F4A6P4	Q0TM08	Q6G060	
	UPI000225F0DB	A9NEN9	B7A901	C4GC72	D1Y0M6	D7BBU4	E4M8X4	F4LV70	Q164N7	Q6G446	
	UPI00022607E7	A9VTL0	B7ACQ7	C4WJD1	D2BI87	D8GJX8	E5VAX4	F4R0Y3	Q1GHT8	Q6NEI3	
	UPI0002276EF4	B0CEI4	B7BFP8	C4XGN7	D2L022	D8JYS1	E5WH97	F5IUS4	Q1NAZ6	Q6YRK5	
	UPI000227805B	B0K8G4	B7DUK7	C4Z4Q6	D2NQ90	D8KQG0	E5XQB3	F5L8F7	Q1YIF0	Q72WV3	
	A0K2H5	A4EBU0	B0MZ11	B7GMU9	C4ZDL6	D2Q3W7	D8PBE7	E6JBU5	F5WV63	Q2B100	Q744V6
	A0LN59	A4ITW0	B0SWL1	B7IS18	C5B2T0	D3D6I2	D9QLL0	E6SR46	F6B6R3	Q2CHT7	Q74FE4
	A0NMU9	A4T4P4	B0TE72	B7RGA1	C5D9X6	D3FQG0	D9QUL2	E6TZX6	F6DRE3	Q2G8G3	Q8EKV4
	A0PKH5	A5CY52	B1BC61	B8D1C9	C5VPU2	D3MQ22	D9RVJ3	E6YL93	F6EVL3	Q2J4C4	Q8KAM1
	A0PX87	A5FYN8	B1LYI4	B8FI57	C6ACL8	D3T6V8	D9S187	E6YV04	F7K7Z9	Q2N9S7	Q8R6M1
1RIS	A1B0F8	A5G7R3	B1VIZ8	B8G0J4	C6BVA8	D4H167	D9SPC2	E6YYE9	F7TPB9	Q2NKC3	Q8YFP0
	A1KEM1	A5P854	B1YG99	B8GVN6	C6Q0F6	D4INL0	E0DH67	E7MR22	F7V2Y9	Q2RM72	Q97CX2
	A1RC70	A6CPQ1	B1ZFQ8	B8H841	C6XKQ8	D4IXN7	E0DNZ2	E8PK10	F7V9G0	Q2RXC6	Q9K5N8
	A1THY7	A6FNZ4	B2A456	B9DXR0	C7DFA2	D4JEM6	E0E596	E8RSF0	F7Z743	Q2W5G8	
	A1UP86	A6LIX7	B2GJD8	B9M5U8	C7G9A9	D4RYK1	E0FSW8	F0F8T7	F7ZE68	Q3A317	
	A1US56	A6WWE6	B2IHD1	B9QWA5	C7JGD3	D4W5D4	E0MLE8	F0IYP4	F8CWC0	Q3AG27	
	A3SHM4	A7AD53	B2RIG3	B9Y3G9	C7MS25	D4Z1E9	E0TB35	F0MA34	F8ET97	Q3Z7N7	
	A3U3U0	A7GVN7	B2S9V4	C0FZZ5	C7N449	D5BRI8	E0U034	F1YSN9	F8JDJ5	Q3ZY12	
	A3UJ78	A7HYI2	B3C5Y0	C0XQX7	C8RVC0	D5MK03	E1GYJ5	F1ZDC1	F8X0G2	Q4JSF9	
	A3WBJ7	A7V6Z5	B3QRL5	C0ZA47	C8W050	D5P9S5	E1K1Z3	F1ZZA3	P21468	Q5FU57	
	A3WZ43	A7ZAU9	B4BPI9	C1PE56	C8WV96	D5QH50	E1KMV3	F2F270	P46389	Q5KU69	
	A3XC67	A8HVF1	B4RAF0	C2M8M2	C9MPQ2	D5WXD1	E1QHQ4	F2JRY7	P62666	Q5LR50	
	A4AEU4	A8L8T4	B4WE93	C2WF67	C9RAG9	D5XDR2	E1UUK2	F2N9C9	Q0BPZ0	Q5WAH3	

More than 100 homologous sequences are obtained for each study protein. The average (or standard deviation) of the sequence identity are 63.0% (16.9), 50.5% (13.9), 37.0% (12.3), and 47.3% (15.3) for U1A, ADA2h, S6, and mtAcP, respectively.

Table S2: The ϕ values of S6 circular permutant P54-55 (2KJW)

Mutation	ϕ value
L8A	0.08
Y10A	0.1
V12A	0.17
V19A	0
L22A	0.51
L26A	0.23
V32A	0.15
V35A	0.61
V37A	0.56
V48A	0.57
I50A	0.4
L52A	0.18
L61A	0.05
I68A	0.23
L72A	0.14
V79A	0.07

List of ϕ values reported in the literature.[15] The residue number is renumbered starting from 1 at the N-terminus. Relatively high ϕ values are found from residue 22 to 50.

Table S3: Proteins used to calculate the inter-residue average distance statistics

PDB ID	Description	PDB ID	Description
2APE	Acid proteinase (Endothia parasitica)	4LDH	Lactate dehydrogenase (dogfish)
2ADK	Adenylate kinase (pig, muscle)	6LYZ	Lysozyme (hen egg white)
4ADH	Alcohol dehydrogenase (horse liver, apo)	1LZM	Lysozyme (bacteriophage T4)
1ABP	L-Arabinose binding protein	1MBN	Myoglobin
2ATC	Aspartate carbamoyl transferase, catalytic subunit	1NXB	Neurotoxin B
1CAB	Carbonic anhydrase B (human)	4PTI	Pancreatic trypsin inhibitor (bovine)
5CPA	Carboxypeptidase A (bovine)	8PAP	Papain
3CAT	Catalase (beef liver)	3PGK	Phosphoglycerate kinase (yeast)
ICHG	Chymotrypsinogen A	3PGM	Phosphoglycerate mutase
3CNA	Concanavalin A	1BP2	Phospholipase A2 (bovine)
1CRN	Crambin	2PAB	Prealbumin (human plasma), A chain
2B5C	Cytochrome b5 (oxidized)	1RHD	Rhodanese
3CYT	Cytochrome c (albacore oxidized), inner	4RSA	Ribonuclease A
4DFR	Dihydrofolate reductase (Lactobacillus casei), B chain	3RXN	Rubredoxin
1FDX	Ferredoxin	ISN3	Scorpion neurotoxin (variant 3)
3FXN	Flavodoxin (Clostridium mp., oxidized)	2SNS	Staphylococcal nuclease
2GRS	Glutathione reductase (human)	1SBT	Subtilisin BPN'
1GPD	D-Glyceraldehyde 3-phosphate dehydrogenase (lobster), green subunit	2SSI	Subtilisin inhibitor (Streptomyces)
1HIP	High potential iron protein (oxidized)	3TLN	Thermolysin
1REI	Immunoglobulin B-J, V-dimer Rei, A chain	1SRX	Thioredoxin reductase
3FAB	h-ImmunoglobulinFab' (heavy chain)	1TIM	Triose phosphate isomerase, A chain

Supporting Information: Figures

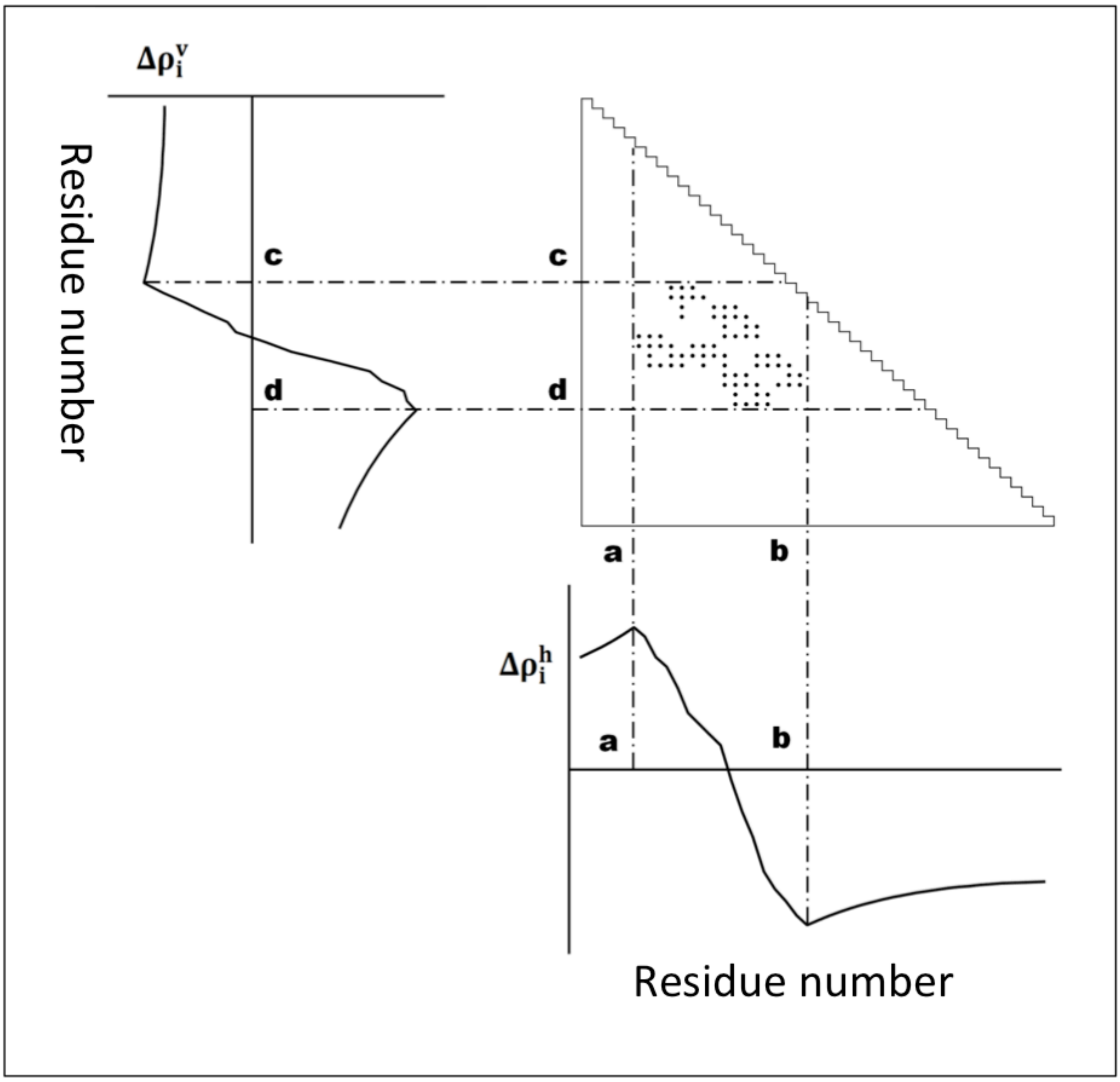

(Figure S1)

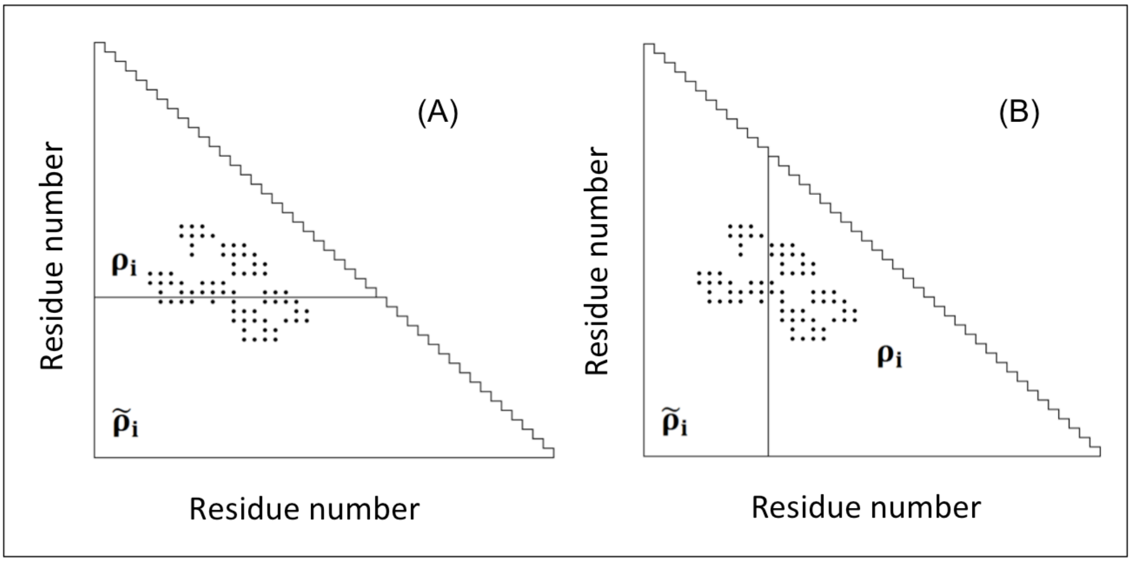
(A)
Residue number
ρ_i
$\tilde{\rho}_i$
Residue number
(B)
Residue number
ρ_i
$\tilde{\rho}_i$
Residue number

(Figure S2)

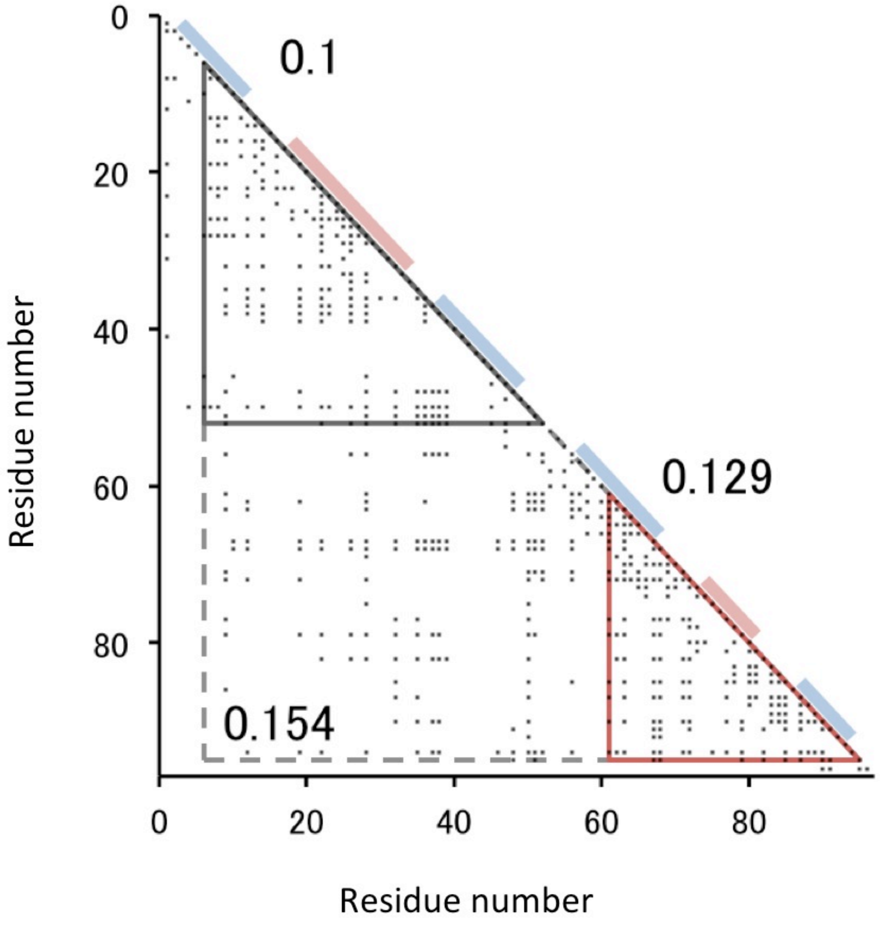
0.1
0.129
0.154
Residue number
Residue number
0
20
40
60
80
0
20
40
60
80

(Figure S3)

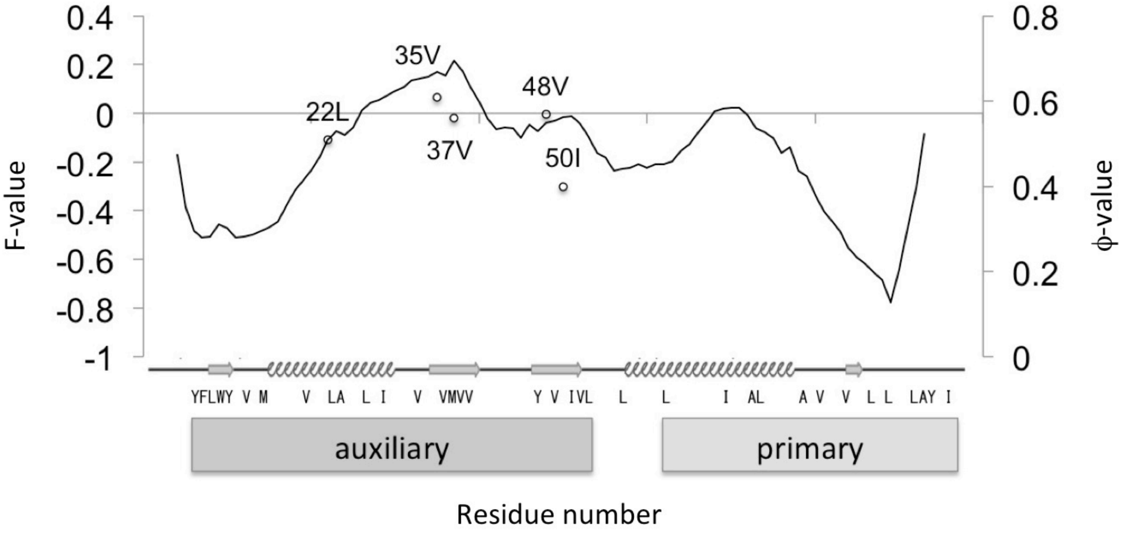

0.4
0.2
0
-0.2
-0.4
-0.6
-0.8
-1
F-value
0.8
0.6
0.4
0.2
0
ϕ-value
35V
22L
48V
37V
50I
YFLWY V M V LA L I V VMVV Y V IVL L L I AL A V V L L LAY I
auxiliary
primary
Residue number

(Figure S4)

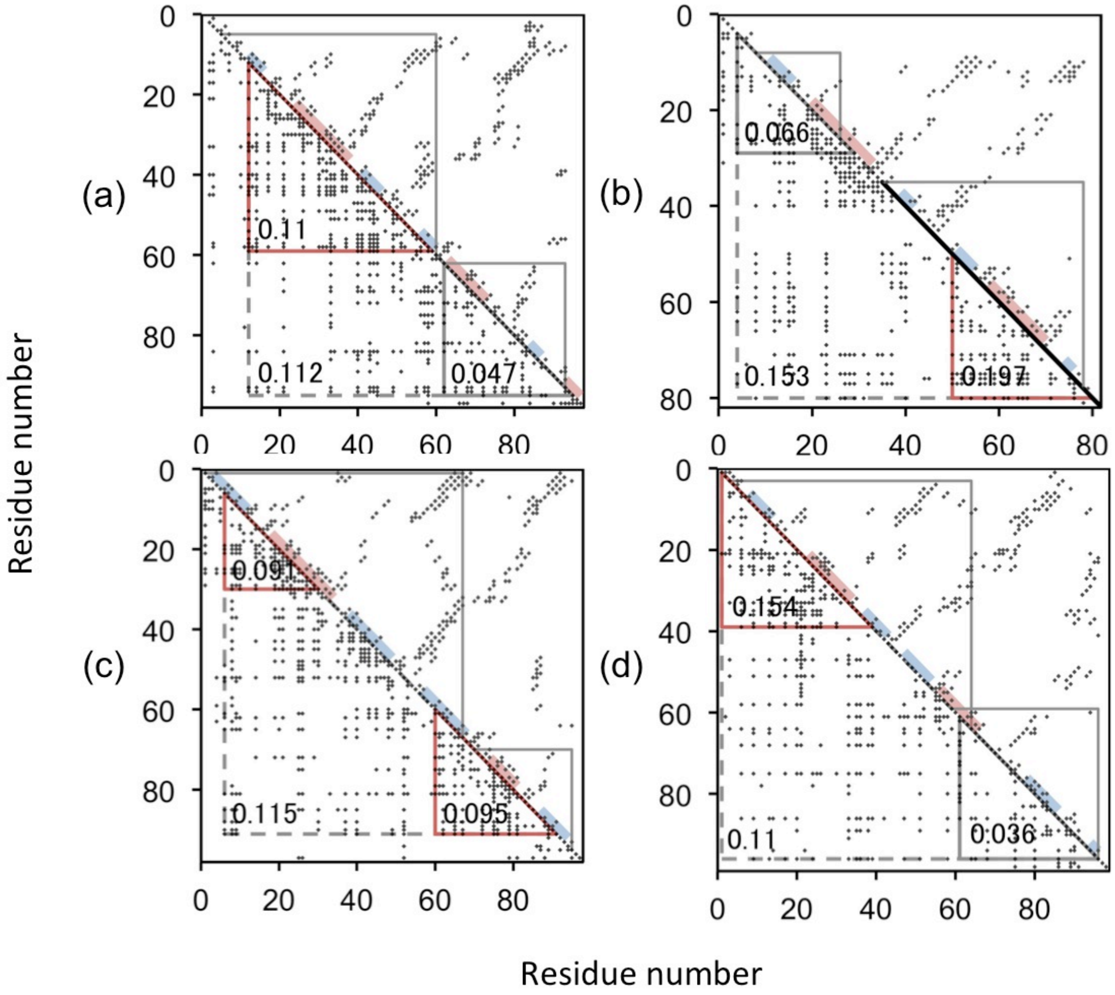
(a)
(b)
(c)
(d)
Residue number
Residue number
0.11
0.112
0.047
0.066
0.153
0.197
0.091
0.115
0.095
0.154
0.11
0.036

(Figure S5)

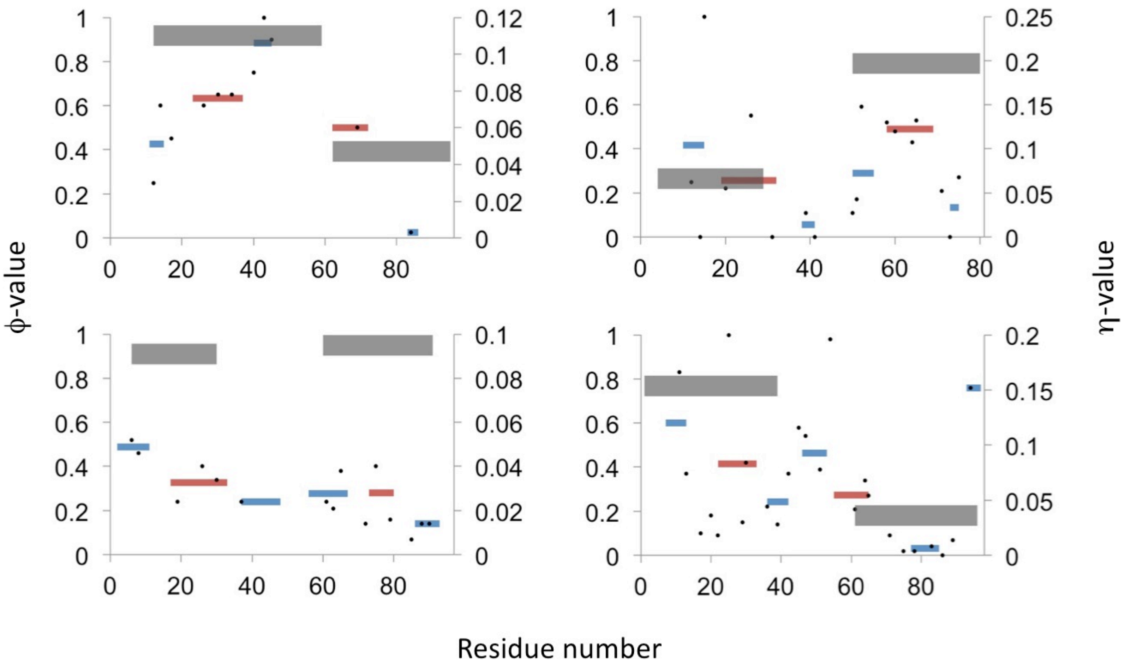

(Figure S6)

(a)

(b)

(c)

(d)

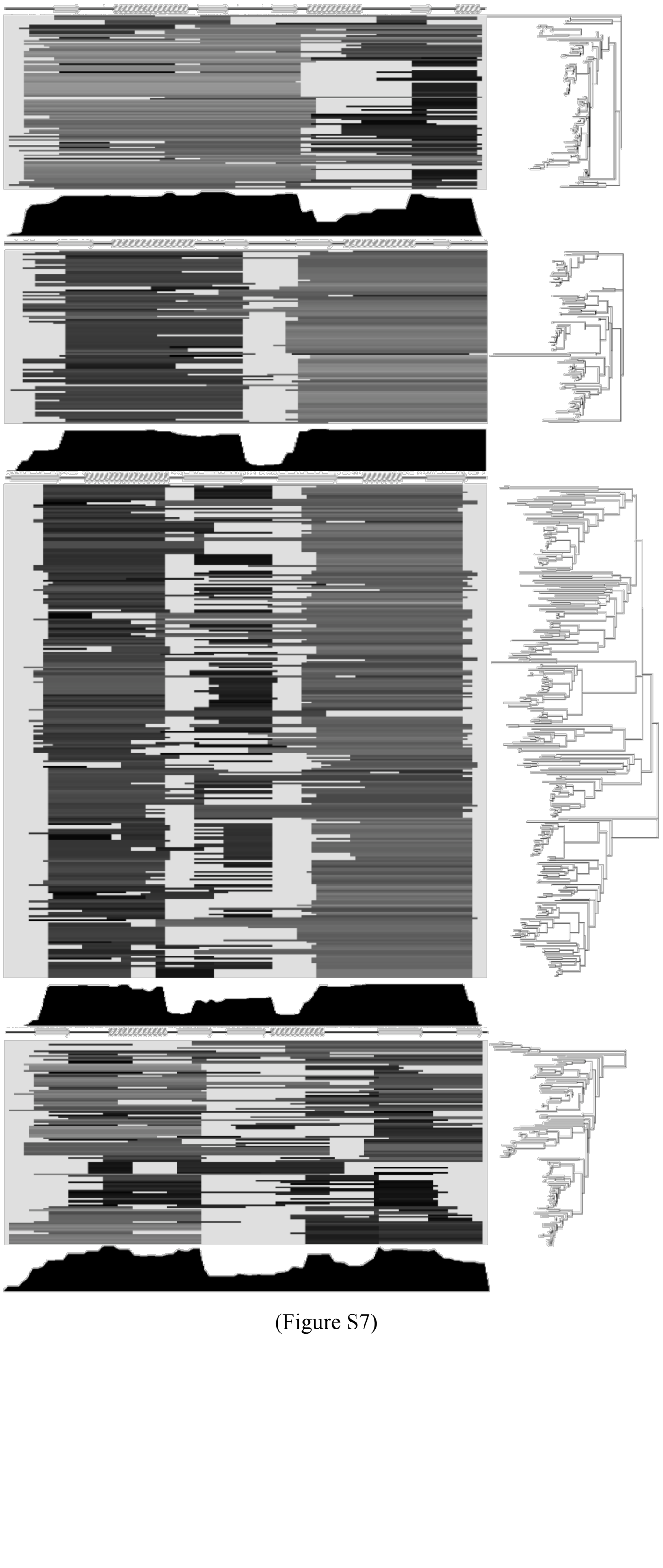

(Figure S7)

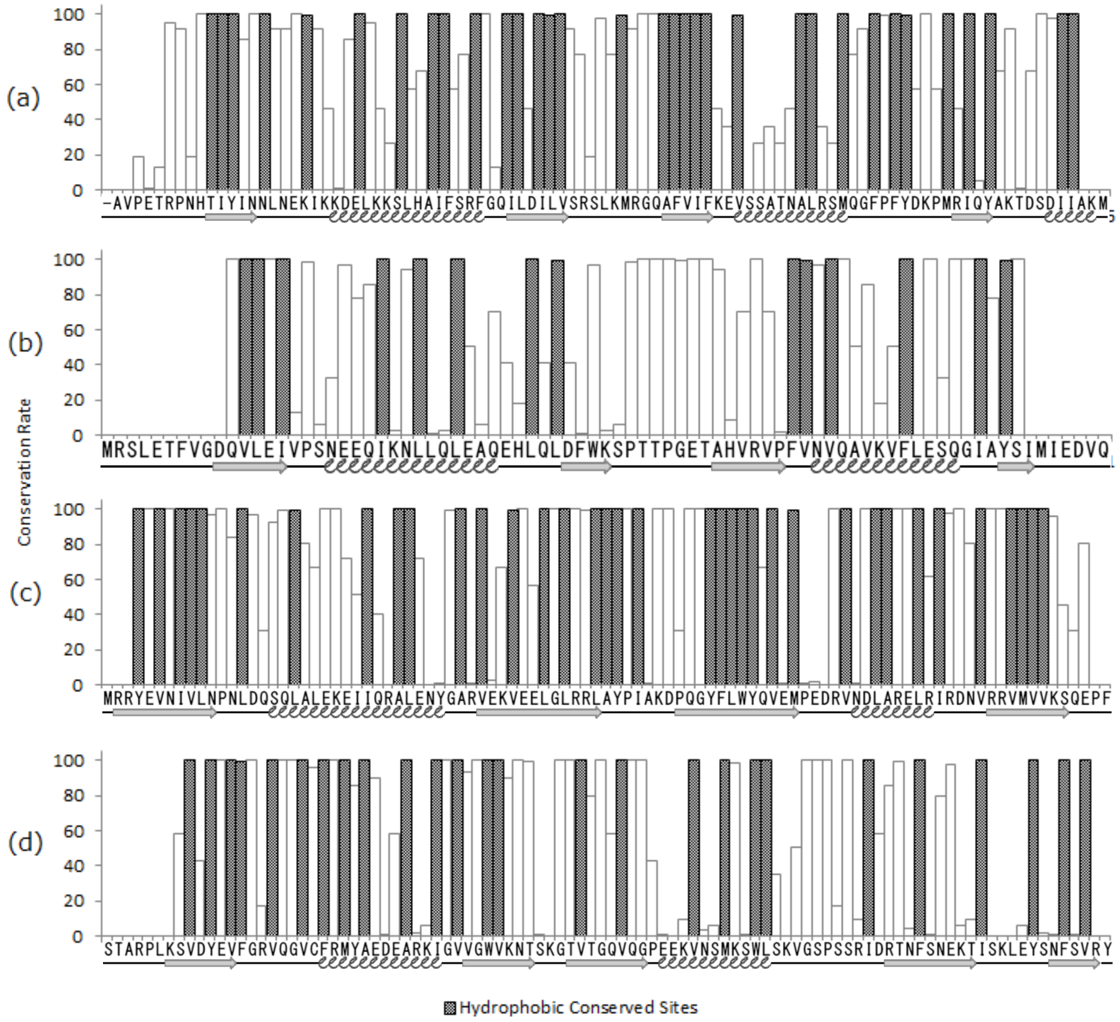

(a)
-AVPETRPNHTIYINNLNEKIKKDELKKSLHAIFSRFGQILDILVSRSLKMRGQAFVIFKEVSSATNALRSMQGFPFYDKPMRIQYAKTDSDIIAKM
(b)
MRSLETFVGDQVLEIVPSNEEQIKNLLQLEAQEHLQLDFWKSPTTPGETAHVRVPFVNVQAVKVFLESQGIAYSIMIEDVQ
(c)
MRRYEVNIVLNPNLDQSQLALEKEIIQRALENYGARVEKVEELGLRRLAYPIAKDPQGYFLWYQVEMPEDRVNDLARELRIRDNVRRVMVVKSQEPF
(d)
STARPLKSVDYEVFGRVQGVCFRMYAEDEARKIGVVGWVKNTSKGTVTGQVQGPEEKVNSMKSWLSKVGSPSSRIDRTNFSNEKTISKLEYSNFSVRY
Conservation Rate
0
20
40
60
80
100
Hydrophobic Conserved Sites

(Figure S8)

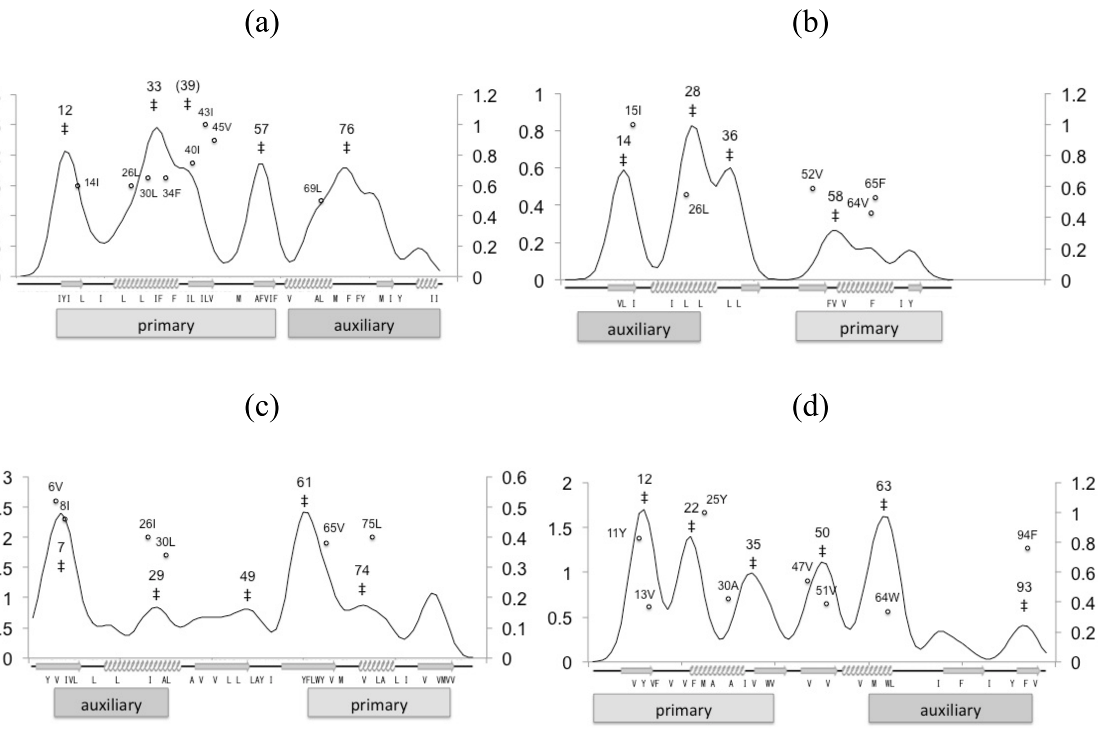
(a)
(b)
(c)
(d)
primary
auxiliary
auxiliary
primary
auxiliary
primary
primary
auxiliary

(Figure S9)

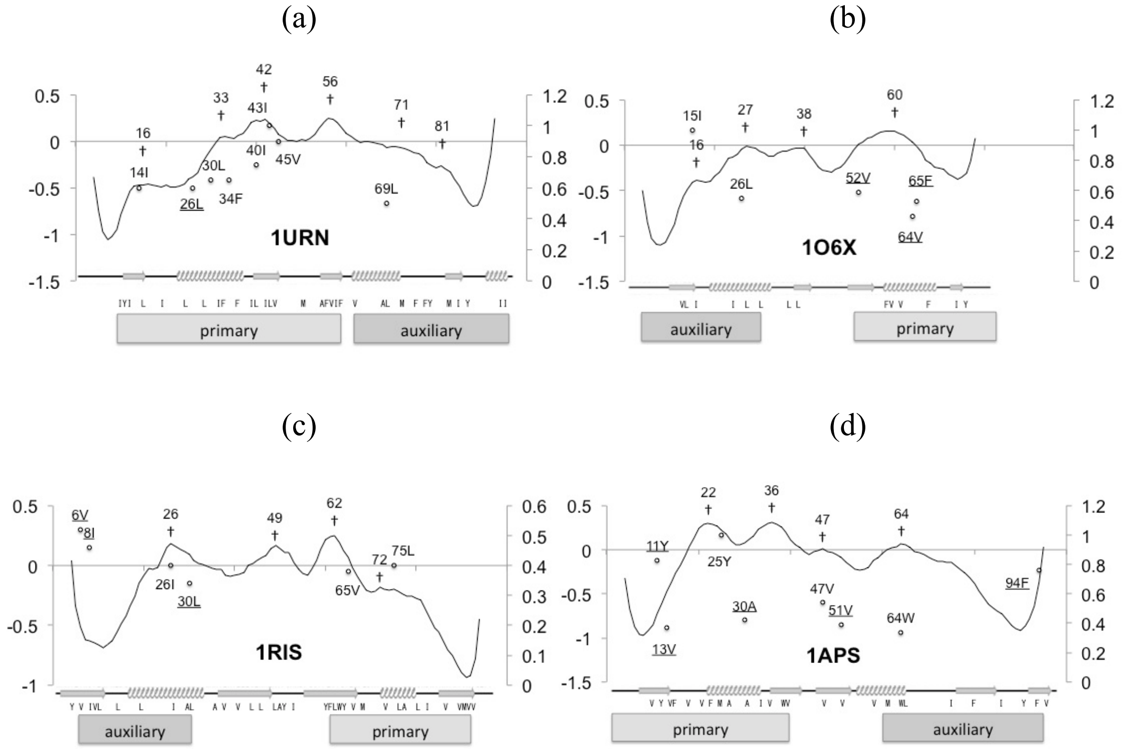
(a)
(b)
(c)
(d)
1URN
1O6X
1RIS
1APS
primary
auxiliary
auxiliary
primary
auxiliary
primary
primary
auxiliary
16
14I
33
42
43I
40I
45V
30L
26L
34F
56
71
69L
81
15I
16
27
26L
38
52V
60
65F
64V
6V
8I
26
26I
30L
49
62
65V
72
75L
11Y
13V
22
25Y
30A
36
47
47V
51V
64
64W
94F

(Figure S10)
